# Elevated levels of sphingolipid MIPC in the plasma membrane disrupt the coordination of cell growth with cell wall formation in fission yeast

**DOI:** 10.1371/journal.pgen.1010987

**Published:** 2023-10-04

**Authors:** Alaina H. Willet, Marcin Wos, Maya G. Igarashi, Liping Ren, Lesley A. Turner, Kathleen L. Gould

**Affiliations:** Department of Cell and Developmental Biology, Vanderbilt University School of Medicine, Nashville, United States of America; University of California San Francisco, UNITED STATES

## Abstract

Coupling cell wall expansion with cell growth is a universal challenge faced by walled organisms. Mutations in *Schizosaccharomyces pombe css1*, which encodes a PM inositol phosphosphingolipid phospholipase C, prevent cell wall expansion but not synthesis of cell wall material. To probe how Css1 modulates cell wall formation we used classical and chemical genetics coupled with quantitative mass spectrometry. We found that elevated levels of the sphingolipid biosynthetic pathway’s final product, mannosylinositol phosphorylceramide (MIPC), specifically correlated with the *css1-3* phenotype. We also found that an apparent indicator of sphingolipids and a sterol biosensor accumulated at the cytosolic face of the PM at cell tips and the division site of *css1-3* cells and, in accord, the PM in *css1-3* was less dynamic than in wildtype cells. Interestingly, disrupting the protein glycosylation machinery recapitulated the *css1-3* phenotype and led us to investigate Ghs2, a glycosylated PM protein predicted to modify cell wall material. Disrupting Ghs2 function led to aberrant cell wall material accumulation suggesting Ghs2 is dysfunctional in *css1-3*. We conclude that preventing an excess of MIPC in the *S*. *pombe* PM is critical to the function of key PM-localized proteins necessary for coupling growth with cell wall formation.

## Author summary

Many organisms have a cell wall that provides structural support and serves as protection against environmental stresses. At the same time, cell walls are dynamic, needing to be repaired and elaborated on as cells grow and divide. A shared challenge among cell-walled organisms is therefore maintaining the integrity of the cell wall while it is being remodeled. The yeast *Schizosaccharomyces pombe* has a well understood pattern of new cell wall deposition at growing tips during interphase and along the septum at division. We took advantage of this knowledge to study a poorly understood step in cell wall assembly defined by a mutation in the *css1* gene that encodes a sphingomyelinase-like enzyme. In these cells, a step following cell wall precursor synthesis fails and therefore, cells stop elongating. We determined that changes in plasma membrane lipid composition and dynamics accompany this defect and identify putative enzymes that act on cell wall precursors after their synthesis to allow cell growth.

## Introduction

For many organisms, the cell wall is the initial barrier protecting them against environmental stresses such as fluctuations in temperature and oxidative and osmotic conditions. At the same time, it provides structural support and helps to maintain cell shape. The cell wall, however, is not like rigid armor but rather, a dynamic and elastic structure that is constantly being remodeled, particularly during cell growth, and a common challenge faced by all cell-walled organisms is how to coordinate the processes of cell growth and cell wall expansion [1–9].

*Schizosaccharomyces pombe* is a rod-shaped yeast that orchestrates cell wall construction in synchrony with its cell division cycle. At the beginning of the cell cycle, *S*. *pombe* cells lengthen from a single tip, the one which existed before division; later, cells switch to bipolar growth, growing at both old and new tips until mitosis [10]. At mitosis, tip extension ceases, and the cells subsequently divide symmetrically by septation. New cell wall material is produced and incorporated into the pre-existing cell wall only at growing tips during interphase and adjacent to the primary septum during cell division [11–13].

The *S*. *pombe* cell wall is composed of three layers as determined by electron microscopy; there are electron-dense outer and inner layers containing galactomannan, and a central core layer comprised of α1,3-glucans, β1,3-glucans and β1,6-glucans [12,14–16]. The glucans are synthesized by the essential glucan synthases Ags1 [17–19], Bgs1 [20–22], Bgs3 [23], and Bgs4 [24], and the galactomannans by multiple non-essential proteins [25]. The linear α1,3-glucans and β1,3-glucans provide rigidity and contribute to cell shape, while the highly branched β1,6-glucans and galactomannans provide flexibility [26,27]. Proteins comprise another important element of the cell wall and play important roles in its structure and function [28–30]. Many cell wall-associated proteins contain a glycosylphosphatidylinositol (GPI) anchor, or its remnant [9,16,31]. In addition to providing protection from the extracellular environment, the *S*. *pombe* cell wall must be unyielding enough to resist an internal turgor pressure of ~1.5 MPa [32,33].

Though the composition of the *S*. *pombe* cell wall has been well-characterized, it is much less clear how new components are incorporated during cell growth [9,13]. In this study, we provide insight into this question by analyzing why cell wall expansion ceases in *css1-3* mutant cells despite continued and abundant production of all cell wall polysaccharides [34]. Css1 is an inositol phosphosphingolipid phospholipase C that catabolizes the complex sphingolipids inositol phosphorylceramide (IPC) and mannosylinositol phosphorylceramide (MIPC) to produce ceramides [34]. We determined by mass spectrometry-based lipidomics and genetic analyses that MIPC accumulation in *css1-3* mutant cells underlies the cell wall defect, in which components used to expand the cell wall collect between the plasma membrane (PM) and the existing cell wall, rather than being incorporated into new cell wall. This situation causes growth arrest, compression of the cytoplasm, and cell death. We used pre-existing and newly developed fluorescent biosensors to show the coordinate accumulation of sphingolipids and sterols in the cytoplasmic PM leaflet in *css1-3* mutant cells. Fluorescence recovery after photobleaching (FRAP) analysis showed that these changes correlated with reduced PM fluidity. We found that blocking the protein N-linked glycosylation machinery globally but not the production of GPI-anchored proteins led to phenotypes similar to those of *css1-3*. Remarkably, mutations in a previously uncharacterized protein, Ghs2, predicted to be glycosylated and involved in cell wall assembly, also partially phenocopied *css1-3*. Our data establish that proper PM sphingolipid levels play a key role in the localization of PM sterols, the properties of the PM, and the function of *S*. *pombe* PM-localized enzymes necessary for coordinating cell wall construction with cell growth.

## Results

### Css1 mutant cells cease cell wall expansion despite ongoing extrusion of cell wall components

**Fig 1:**
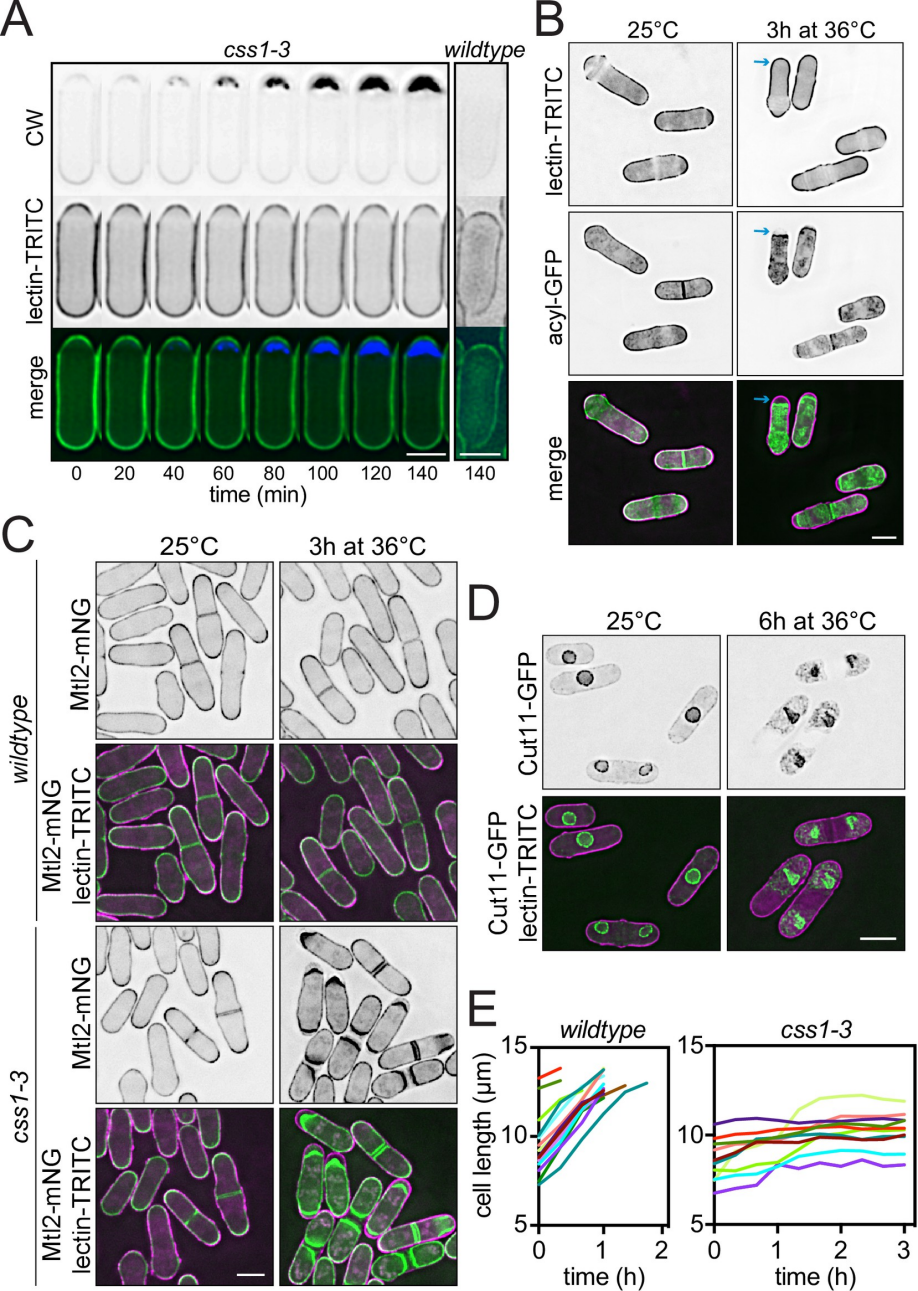
Characterization of the *css1-3* phenotype. (A) Representative montage from live-cell, time-lapse imaging of a *css1-3* cell grown 25°C and shifted to 36°C for 3 h. Cells were treated with TRITC-lectin for 10 min and with calcofluor white (CW) for 5 min before time = 0. A representative 140-minute time point of a wildtype cell grown and imaged under the same conditions is also shown. (B) Live-cell imaging of *css1-3* cells expressing *acyl-GFP* grown at 25°C and shifted to 36°C for 3 h. Cells were treated with TRITC-lectin for 10 minutes before imaging. Blue arrows indicate where cell wall material accumulated in that cell. (C) Live-cell imaging of wildtype and *css1-3* cells expressing *mtl2-mNG* grown at 25°C and then shifted to 36°C for 3 h. Cells were treated with TRITC-lectin for 10 minutes before imaging. (D) Live-cell imaging of *css1-3 cut11-GFP* cells grown at 25°C then shifted to 36°C for 6 h. Cells were treated with TRITC-lectin for 10 minutes before imaging. (E) Wildtype and *css1-3* cells were grown at 25°C, shifted to 36°C for 3 h and imaged using differential interference contrast (DIC). Cell length was measured and plotted against time. n = 10. Scale bars, 5 μm.

Temperature-sensitive recessive mutations in the *S*. *pombe css1* gene lead to elevated levels of glucans and galactomannans [34]. The accumulation of this material underneath the previously existing cell wall was visualized by live-cell imaging of *css1-3* after shifting to the non-permissive temperature with the addition of calcofluor white (CW), which binds glucans, and TRITC-conjugated lectin, which labels the exterior of the cell wall (Fig 1A). Imaging *css1-3* cells expressing acylated GFP (acyl-GFP) as a marker of the PM showed that glucans were deposited between the PM and the cell wall at the restrictive temperature (Fig 1B). A marker of the PM at cell tips and septa, the mechanosensitive transmembrane protein Mtl2 [35], tagged with mNeonGreen (mNG), also accumulated adjacent to the glucan deposits but displaced away from the cell wall (Fig 1C).

Despite the accumulation of cell wall constituents in the periplasmic space, *css1-3* cells maintained their rod-shaped morphology while the cytoplasm appeared to become compressed (Fig 1A–1C) [34]. Additional observations using the nuclear envelope marker Cut11-GFP [36] showed significant deformation of the nucleus (Fig 1D), in line with cytoplasmic compression. *css1-3* mutant cells also appeared to cease tip extension at the non-permissive temperature [34]. We validated this observation by tracking growth of individual cells over time. Whereas wildtype cells shifted to 36°C grew to 14 ± 0.52 μm before septating, *css1-3* cells ceased elongation within an hour of incubation at 36°C (Fig 1E).

Because *css1-3* cells remained rod-shaped and cell wall components were extruded only at tips and/or septa where new cell wall is normally constructed, we predicted that the glucan synthases Ags1, Bgs1, Bgs3, and Bgs4 would localize normally and adjacent to the glucan deposits in *css1-3* mutant cells. Indeed, all four proteins localized at tips and septa of *css1-3*, as in wildtype cells (S1A Fig) [23,24,27,37,38]. We further reasoned that reducing the extent of cell wall precursor synthesis might suppress the *css1-3* growth defect. As predicted, the conditional alleles alone or in combination (*ags1-664*, *bgs1-191* and/or *bgs4-1* of α1,3-glucan synthase, linear β1,3-glucan synthase and 1,6 branched β1,3-glucan synthase, respectively) [18,24,39], improved *css1-3* cell growth at semi-permissive temperatures (S1B Fig) while growth in hypoosomotic conditions (sorbitol-containing media) did not (S1C Fig).

Over-production of Wsc1, a cell wall stress sensor, activates the cell integrity pathway (CIP) and Rho1 to stimulate glucan synthase activity [35,40,41]. We therefore hypothesized that eliminating CIP function would reduce glucan synthase activity in *css1-3* cells and improve their growth. Indeed, the deletion of *wsc1Δ* and many CIP components including *rho2Δ*, *pmk1Δ*, *rgf1Δ*, *mkh1Δ*, *pek1Δ*, *pck1Δ*, and *pck2Δ* partially suppressed *css1-3* lethality (S2A Fig). This is in accord with Wsc1-mNG forming large clusters in *css1-3* cells (S2B Fig), indicative of CIP activation [42]. Taken together, our data indicate that mutations in *css1* block a step in cell wall assembly downstream of the production and extrusion of cell wall constituents into the extracellular space.

### Preventing synthesis of MIPC suppresses the *css1-3* mutant phenotype

**Fig 2:**
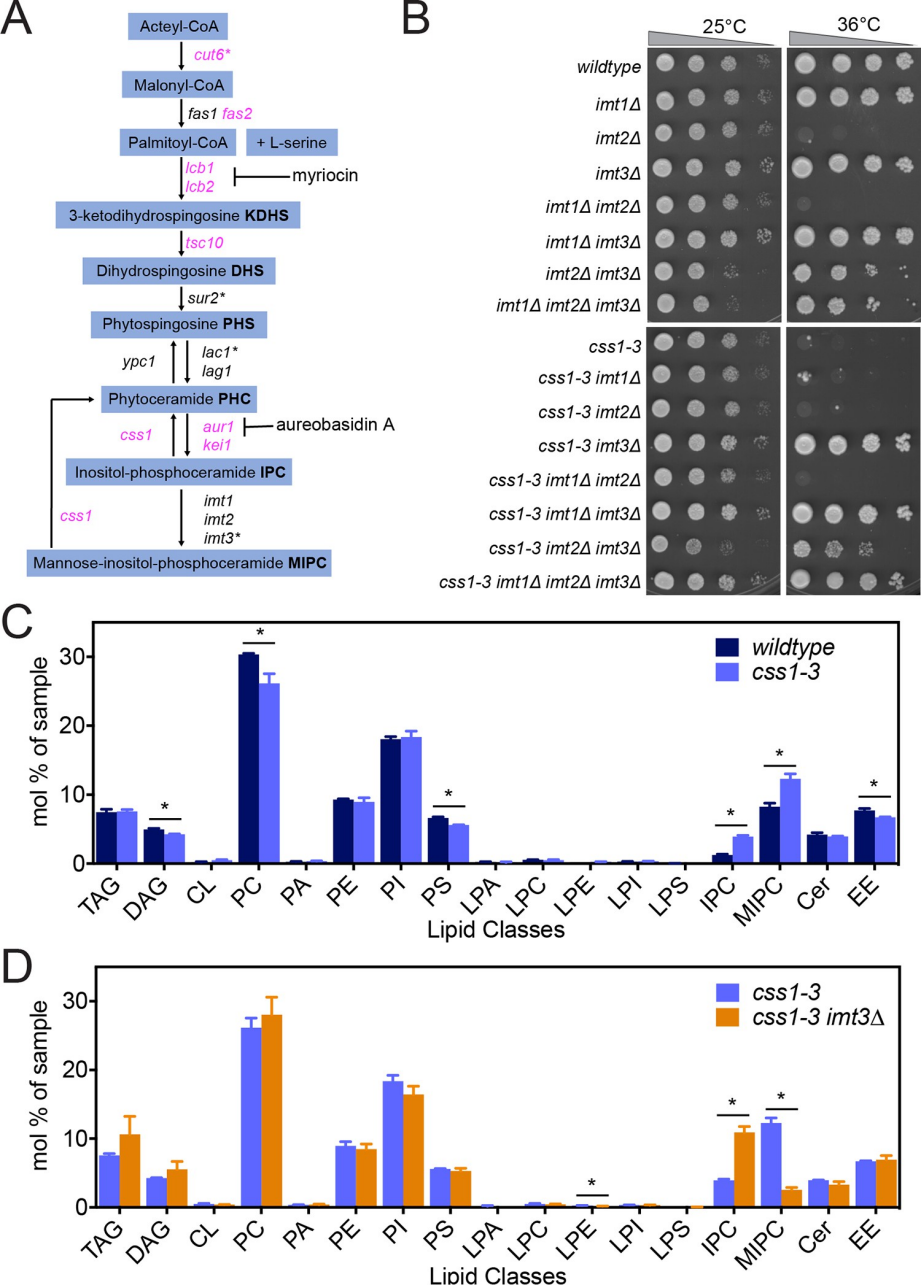
*css1-3* cells accumulate MIPC at non-permissive temperature. (A) A simplified schematic of the sphingolipid synthesis pathway in *S*. *pombe*. Genes in black text are nonessential; genes in magenta text are essential. Asterisks indicate the genes whose inhibition suppresses c*ss1-3*. The steps inhibited by myriocin and aureobasidin A are indicated. (B) Serial 10-fold dilutions of the indicated strains were spotted on YE plates and incubated at the indicated temperatures for 3 days. (C-D) Wildtype, *css1-3*, and *css1-3 imt3∆* cells were grown in YE media at 25°C, shifted to 36°C for 1 h, and lipids were extracted and analyzed as described in Materials and Methods. In C, the percentage of each lipid class in wildtype and *css1-3* cells is shown. In D, the sphingolipid profile of *css1-3* is compared to *css1-3 imt3Δ*. TAG, triacylglycerol; DAG, diacylglycerol; CL, cardiolipin; PC, phosphatidylcholine; PA, phosphatidic acid; PE, phosphatidylethanolamine; PI, phosphatidylinositol; PS, phosphatidylserine; LPA, lyso-phosphatidate; LPC, lyso-phosphatidylcholine; LPE, lyso-phosphatidylethanolamine; LPI, lyso-phosphatidylinositol; LPS, lyso-phosphatidylserine; IPC, inositolphosphorylceramide; MIPC, mannosyl-inositol-phosphorylceramide; CER, ceramide (phytoceramide + dihydroceramide); EE, ergosteryl ester.

The predicted substrates of Css1 are the last two products of sphingolipid biosynthesis, IPC and MIPC [34,43]. Therefore, we reasoned that the *css1-3* phenotype could result from an accumulation of MIPC, IPC, and/or any upstream intermediate(s) in the sphingolipid biosynthetic pathway (Fig 2A). To identify which sphingolipid or sphingolipid precursor was linked to the *css1-3* phenotype, we used conditional and deletion mutants of genes in the sphingolipid synthesis pathway, as well as chemical inhibition of pathway enzymes. Cut6, acetyl CoA carboxylase, acts at the top of the pathway so disruption of its function inhibits the production of all intermediates needed for sphingolipid biosynthesis [44]. Indeed, we isolated a cold-sensitive mutant of *cut6*, *cut6-1*, as a strong suppressor of *css1-3* (S3A Fig). Myriocin, which inhibits ER enzymes Lcb1 and Lcb2 to block synthesis of sphingolipid and ceramide precursors [45,46], also fully suppressed the *css1-3* growth defect (S3B Fig). ER-localized Sur2 is a sphingosine hydroxylase involved in the biosynthesis of phytosphingosine and phytoceramides and *sur2∆* causes an accumulation of IPC and a decrease in MIPC levels [47,48]. *sur2∆* suppressed *css1-3* (S3C Fig). Lac1 and Lag1 are ER-localized ceramide synthases that produce distinct species of ceramides and have different effects on cell growth [49]. Lac1 is proposed to be particularly important for MIPC production, and both are important for IPC production [49]. *lac1Δ* suppressed *css1-3* but *lag1Δ* did not (S3D Fig). A non-lethal concentration of aureobasidin A, which inhibits the essential Golgi enzyme Aur1 and limits the synthesis of IPC [50–52] also suppressed *css1-3* (S3B Fig).

Finally, *imt3∆* strongly suppressed *css1-3* growth (Fig 2B) and the accumulation of cell wall material (S3E Fig). Imt3 is one of three highly related mannosyltransferases (the other two being Imt1 and Imt2) acting at the Golgi in the final step of the biosynthetic pathway to catalyze the formation of the five subspecies of MIPC from IPC [47]. Interestingly, neither *imt1∆* nor *imt2∆* suppressed *css1-3* (Fig 2B), in line with the idea that the three mannosyltransferases have distinct catalytic activities toward IPC subspecies and/or distinct IPC subspecies as substrates. Relevant to this possibility, we noticed that the *imt2∆ and imt1∆ imt2∆* strains were temperature sensitive (Fig 2B). Microscopic analysis revealed that, as in *css1-3*, cell wall material accumulated at the cell division site in *imt2∆* cells (S3F Fig). This phenotype was suppressed by *imt3Δ* (S3F Fig). These results suggest that in the absence of Imt2, Imt3 may become the predominant mannosyltransferase and Imt3-specific MIPC subspecies may increase in *imt2∆* strains. These data also suggest that Imt3-dependent MIPC subspecies are particularly important to regulate. Collectively, our genetic results suggest that the *css1-3* phenotype results from an accumulation of the final product of the sphingolipid biosynthetic pathway, MIPC.

To define more precisely which lipid species drive the *css1-3* phenotype, quantitative mass spectrometry (MS)-based lipidomic analysis of wildtype, *css1-3*, *imt3∆*, and *css1-3 imt3∆* cells grown at 25°C in YE and shifted for 1 hour to 36°C was performed. A comparison of the lipid profile of *css1-3* to that of wildtype showed decreased levels of diacylglycerol (DAG), phosphatidylcholine (PC), phosphatidylserine (PS) and ergosterol esters (EE) (Fig 2C). *css1-3* cells also showed an increase in the levels of IPC and MIPC relative to wildtype (Fig 2C). The suppressed double mutant, *css1-3 imt3∆*, had an even higher level of IPC than *css1-3* alone. However, the level of MIPC was lower in *css1-3 imt3∆* cells than in wildtype (Fig 2D). Altogether, these quantitative MS results support the genetic epistasis analysis and indicate that it is MIPC accumulation that underlies the cellular defects of *css1-3*.

### Css1 is a PM-localized protein

**Fig 3:**
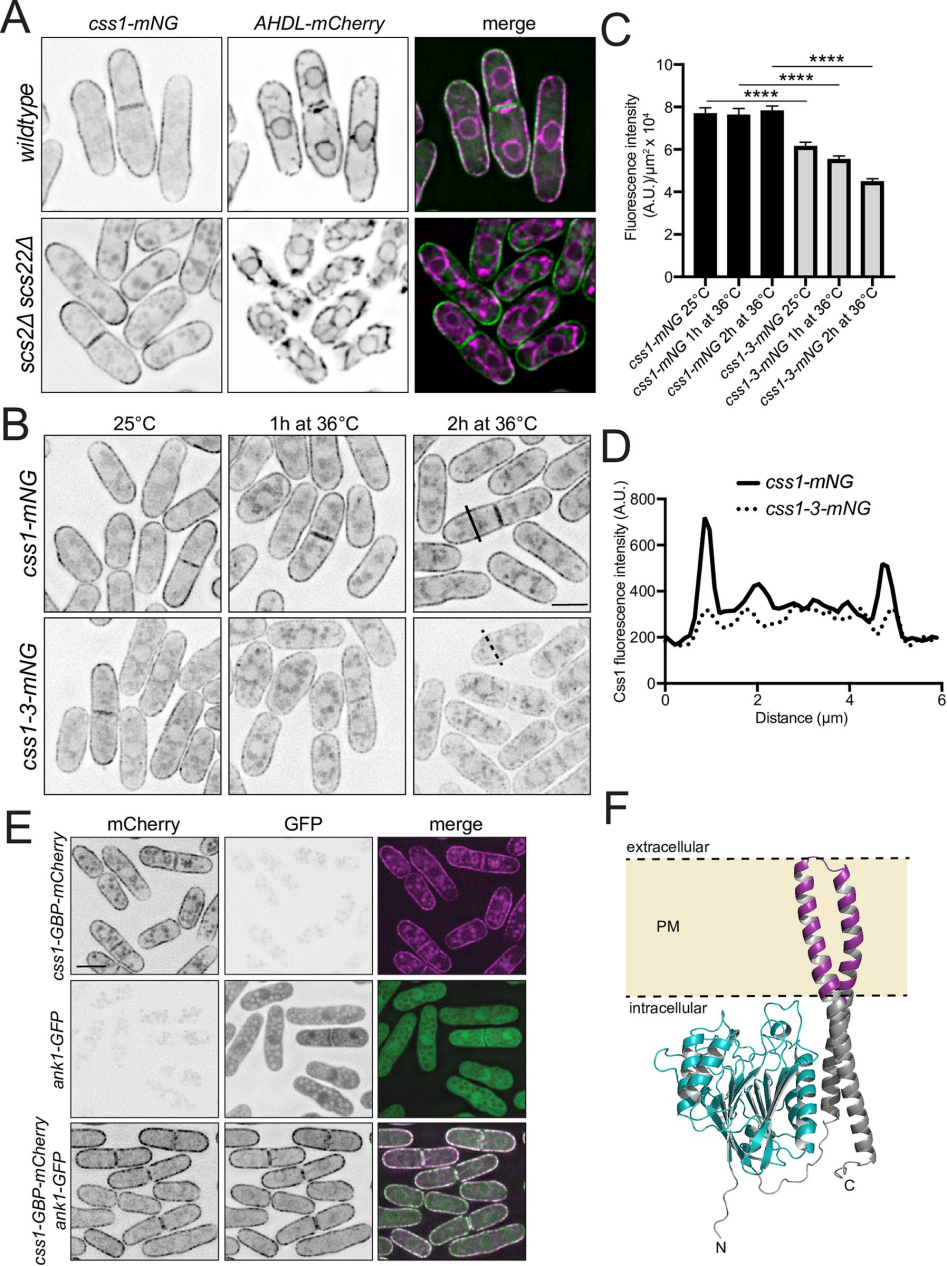
Css1 is a PM protein. (A) Live-cell imaging of *css1-mNG* and *AHDL-mCherry* in wildtype and *scs2∆ scs22∆* cells. (B) Live-cell imaging of *css1-mNG* and *css1-3-mNG* at 25°C and after shifting to 36°C for 1 and 2 h. (C) Quantification of fluorescence intensity per μm^2^ of whole non-septated cells from B. n ≥ 45 cells for each strain. Error bars represent SEM. ****p ≤ 0.0001; one-way ANOVA (D) Line scans of fluorescence intensity across the short axis of *css1-mNG* (solid black line) and *css1-3-mNG* (dotted black line) indicated in the 2 h time point in B. (E) Live-cell imaging of *css1-GBP-mCherry*, *ank1-GFP* and *css1-GBP-mCherry ank1-GFP* cells grown up at 25°C. (F) AlphaFold predicted structure of Css1.The two transmembrane domains are in magenta and the Css1 catalytic phospholipase domain is in cyan. Css1 N and C termini are also labeled. Scale bars, 5 μm.

Biosynthesis of sphingolipids begins in the endoplasmic reticulum (ER) and is completed in the Golgi complex, but sphingolipids ultimately localize primarily in the PM, where they are thought to reside mostly in the outer leaflet [53–57]. Overexpressed GFP-Css1 localizes at the cell periphery [34]. Because the cortical ER underlies the PM in *S*. *pombe* [58], we asked whether Css1 was a PM or cortical ER-localized enzyme. For this, Css1 was tagged with sequences encoding mNG at its endogenous locus in a strain that also produced the ER marker, mCherry-AHDL [59]. Endogenous Css1-mNG was detected only at the cell periphery and did not appear to co-localize significantly with mCherry-AHDL (Fig 3A). To conclusively distinguish peripheral ER from PM localization, we examined Css1-mNG in *scs2∆ scs22∆* cells in which ER-PM contact sites are disrupted and the ER is partially separated from the PM [60]. Css1-mNG localization remained cortical in areas where the ER was detached from the PM (Fig 3A). We conclude that Css1 is a bona fide PM protein. We next examined the localization of the mutant Css1-3-mNG protein as cells were shifted to the non-permissive temperature. We found that the amount of Css1-3-mNG declined over time (Fig 3B–3C). Moreover, it was lost specifically from the PM (Fig 3B and 3D). Taken together, we conclude that it is likely a failure to catabolize complex sphingolipids at the PM that leads to their over-abundance in *css1-3* mutant cells.

Css1 lacks a signal sequence and is predicted to have two C-terminal transmembrane domains [61–63]. These likely form a hairpin loop that inserts into the PM such that both protein termini and the catalytic phospholipase domain would be positioned intracellularly [34,64]. To confirm this, we tagged Css1 with sequences encoding GFP-binding protein (GBP)-mCherry in cells producing a cytoplasmic GFP tagged protein, Ank1-GFP. We reasoned that if the Css1 C-terminus is intracellular, cytoplasmic Ank1-GFP would be recruited to the PM but if extracellular, it would not be accessible to the GFP-tagged protein. In cells expressing Css1-GBP-mCherry Ank1-GFP, Ank1-GFP was indeed recruited to the PM (Fig 3E). Therefore, we conclude that the catalytic domain of Css1 is positioned at the inner leaflet of the PM to control MIPC levels (Fig 3F).

### Localization of PM sterols is altered in *css1*-*3*

The biophysical properties of sphingolipids, namely their large hydrophilic heads and saturated acyl chains, allows them to interact with sterols in model membranes [65], in which they form PM microdomains [66–69]. There is also genetic evidence that these two classes of lipids function together in *Saccharomyces cerevisiae* [70]; mutants defective in ergosterol or sphingolipid biosynthesis affect the other’s synthesis and membrane composition, suggesting that the production and deployment of these two lipids is coordinated [70–72]. Although we did not detect a significant change in sterol levels in *css1-3* cells by MS (Fig 2C), we tested whether the intracellular distribution of membrane sterols was altered in *css1-3* cells.

**Fig 4:**
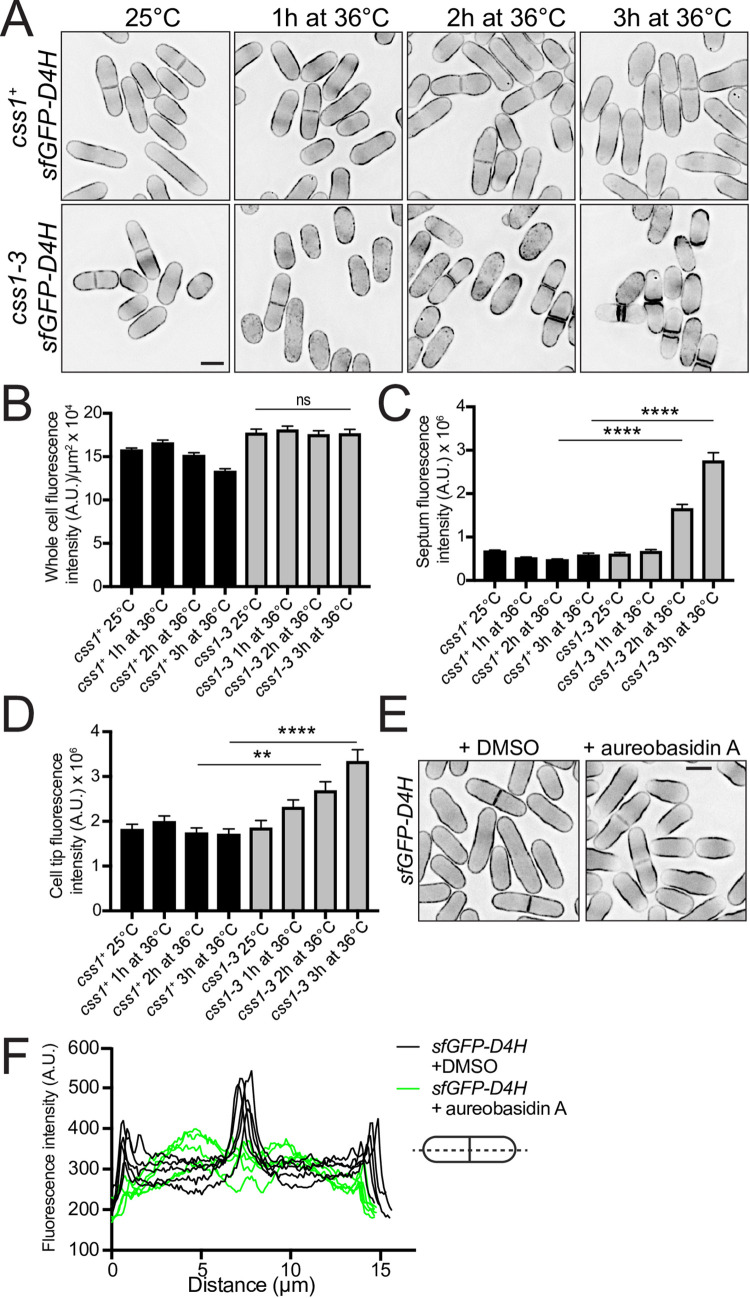
Sterol distribution is altered in *css1* mutant cells. (A) Live-cell imaging of wildtype and *css1-3* cells expressing sterol sensor *sfGFP-D4H* that were grown at 25°C then shifted to 36°C and imaged each hour. (B) Quantification of fluorescence intensity per μm^2^ of whole non-septated cells from A. n = 45. *css1-3* 25°C vs. *css1-3* 3h at 36°C, p > 0.99. (C) Quantification of septum intensity of cells from A. n ≥ 29. (D) Quantification of cell tip intensity of cells from A. n ≥ 83. For B, C and D error bars represent SEM. **p ≤ 0.01, ****p ≤ 0.0001; one-way ANOVA. (E) Live-cell imaging of wildtype cells expressing sterol sensor *sfGFP-D4H* grown at 25°C and treated with methanol or 50 ng/ml aureobasidin A in methanol for 3 h. (F) Five representative line scans across septated *sfGFP-D4H* cells treated with methanol (black lines) or 50 ng/ml aureobasidin A (green lines) from E. Scale bars, 5 μm.

For this, we used a genetically encoded sterol biosensor (DH4-D434S) expressed from the constitutive *act1* promoter linked to two different fluorophores (mCherry and sfGFP) [73–76]. In wildtype cells at 25°C and 36°C, and *css1-3* cells at 25°C, both probes localized to the cell periphery and were also detected in cytosolic puncta (Figs 4A and S4A), as expected from previous work [76]. While the whole cell intensities of the probes did not change in *css1-3* cells shifted to 36°C, probe intensities increased adjacent to deposits of cell wall components at the septum and cell tips (Figs 4A–4D and S4A-S4D). Thus, there appears to be a redistribution of intracellular sterols in *css1-3* cells. Reciprocally, treatment of cells with aureobasidin A to block formation of IPC and MIPC resulted in decreased sterol sensor intensities at both cell tips and septa (Fig 4E–4F). We did not detect a difference in filipin staining, which marks sterols in the outer PM leaflet, between wildtype and *css1-3* at either permissive or restrictive temperature (S4E Fig). Collectively, these data indicate that the level of complex sphingolipids controls the distribution of PM sterols, at least within the PM inner leaflet, in *S*. *pombe*.

Based on these observations and the potential of sterol-sphingolipid co-regulation, we hypothesized that reducing sterol formation might suppress *css1-3* by decreasing sphingolipid synthesis or causing a redistribution of PM MIPC. We found that some deletions of genes involved in ergosterol biosynthesis partially suppressed *css1-3* and one, *erg4*, fully suppressed *css1-3* growth and accumulation of cell wall precursors (S4F–S4G Fig). Erg4 (first known as Sts1) is a C-24(28) sterol reductase that acts at the final stage of ergosterol biosynthesis [77,78].

**Fig 5:**
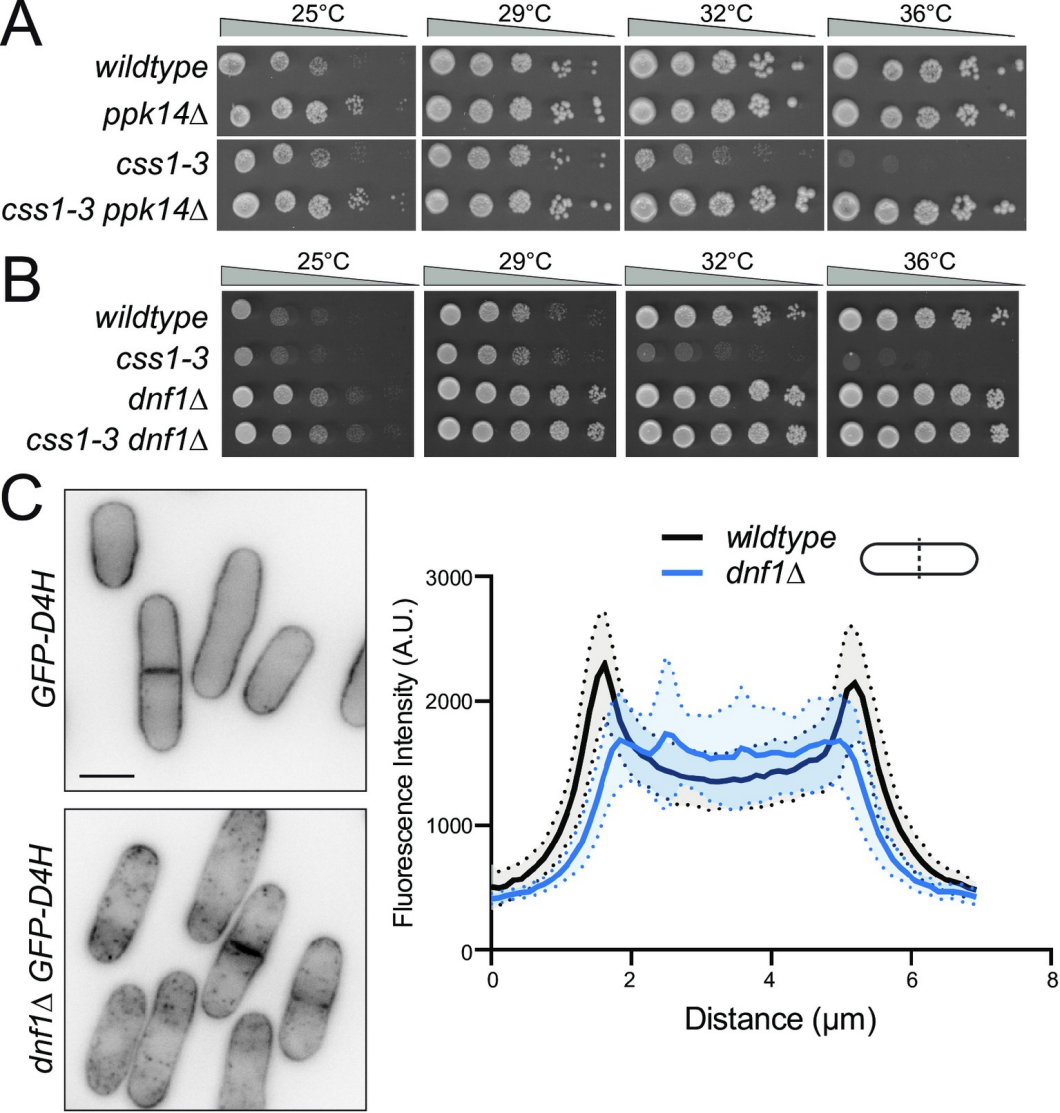
Mutations in the Ppk14 signaling pathway suppress *css1-3*. (A-B) Serial 10-fold dilutions of the indicated strains were spotted on YE plates and incubated at the indicated temperatures. (C) Left, live-cell imaging of wildtype and *dnf1∆* cells expressing sterol sensor *sfGFP-D4H* that were grown and imaged at 25°C. Scale bar, 5 μm. Right, plot of the fluorescence intensity of *sfGFP-D4H* across the short cell axis of wildtype or *dnf1∆* cells. Shaded area represents SEM. n = 10.

Evidence from *S*. *cerevisiae* suggests that MIPC enhances the activity of PM protein kinase Fpk1, which in turn activates PM phospholipid flippases [79,80]. In *S*. *cerevisiae*, phospholipid flippase function is required to retain ergosterol at the PM [81]. This model suggests that elevated PM MIPC levels in *css1-3* cells may lead to hyper-activated Ppk14 (the *S*. *pombe* ortholog of Fpk1), hyper-activated PM phospholipid flippases, and more ergosterol in the PM. If this was the case, we hypothesized that blocking these activities might also rescue *css1-3* lethality. Indeed, *ppk14Δ* and also deletion of the gene encoding phospholipid flippase *dnf1* but not *dnf2Δ* or *dnf2*.*5Δ* [82] robustly suppressed *css1-3* (Figs 5A–5B and S5A). Consistent with functions at the PM, we found that both Ppk14-mNG and Dnf1-moxNG (an oxidizing environment-optimized variant of mNG [83], see Materials and Methods) localized at the PM (S5B Fig). Finally, there was less GFP-D4H sterol sensor at the PM in *dnf1∆* cells (Fig 5C), as predicted from work in *S*. *cerevisiae* [81].

### Localization of PM complex sphingolipids are altered in *css1*-*3*

**Fig 6:**
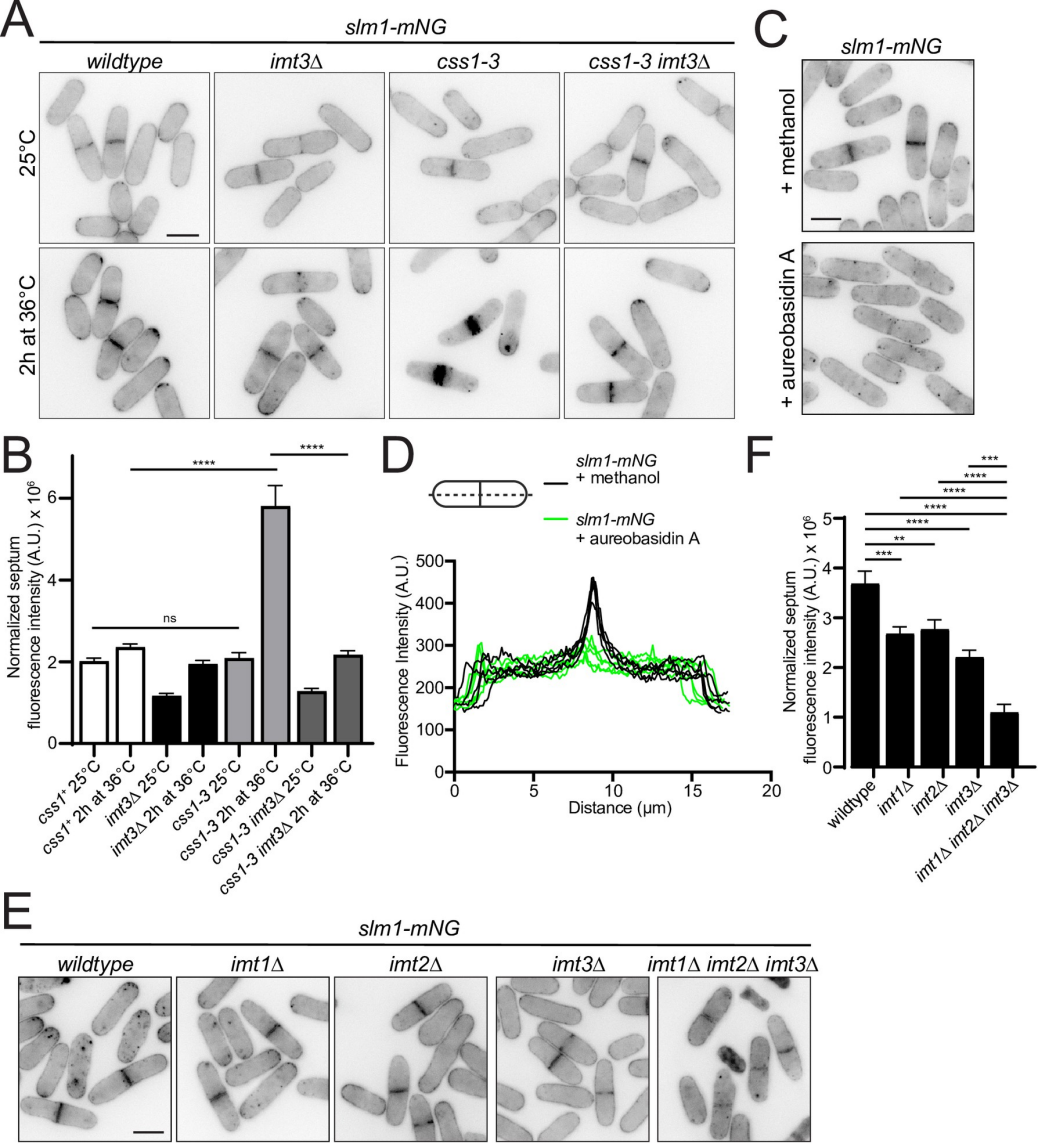
Slm1 is a sensor for complex sphingolipid levels. (A) Live-cell imaging of wildtype, *css1-3*, *imt3Δ* and *css1-3 imt3Δ* cells expressing Slm1-mNG grown at 25°C and then shifted to 36°C for 2 h. (B) Quantification of septum intensity of cells from A. n ≥ 29. Error bars represent SEM. ****p ≤ 0.0001; one-way ANOVA. *css1*^*+*^ 25°C vs *css1-3* 25°C, p > 0.99. (C) Live-cell imaging of wildtype cells expressing Slm1-mNG grown at 25°C and treated with methanol or 50 ng/ml aureobasidin A in methanol for 3 h. (D) Five representative line scans across septated cells treated with methanol (black lines) or aureobasidin A (green lines) from C (E) Live-cell imaging of wildtype, *imt1Δ*, *imt2Δ*, *imt3Δ* and *imt1Δ imt2Δ imt3Δ* cells expressing Slm1-mNG and grown at 25°C (F) Quantification of septum intensity of cells from C. n ≥ 29. Error bars represent SEM. **p ≤ 0.01, ***p ≤ 0.001, ****p ≤ 0.0001; one-way ANOVA. Scale bars, 5 μm.

Examining the localization of membrane sphingolipids is problematic because they are thought to be primarily on the outer leaflet of the PM, and a genetically-encoded sensor is not available for monitoring them in yeast. However, the PH domain of *S*. *cerevisiae* Slm1 was posited to act as a coincidence detector for PM phosphatidylinositol phosphates and sphingolipids, and the PM localization of *S*. *cerevisiae* Slm1-GFP is disrupted when cells are treated with myriocin that blocks ceramide and sphingolipid production [84]. We therefore analyzed the localization of the orthologous protein in *S*. *pombe*, also named Slm1. Slm1 C-terminally tagged with mNG (Slm1-mNG) localized along the PM including at tips and septa, as well as at internal vesicles in wildtype cells at 25°C and 36°C, as previously observed [85] (Fig 6A). In *css1-3* cells at the restrictive temperature, Slm1-mNG accumulated at cell tips and septa adjacent to the deposits of cell wall precursors (Fig 6A–6B). Consistent with it recognizing an increased level of complex sphingolipids there, aureobasidin A treatment decreased Slm1-mNG intensity at the septum relative to wildtype cells (Fig 6C–6D). Thus, *S*. *pombe* Slm1 is sensitive to IPC and/or MIPC levels.

To determine whether Slm1 was sensitive to IPC, MIPC, or both, we imaged Slm1-mNG in strains lacking one or all three mannosyltransferases, Imt1, Imt2, and Imt3. We expected that if Slm1 was sensitive to PM IPC, Slm1-mNG localization to the PM would increase in these strains based on our MS-based lipid profile (Fig 2C–2D), whereas if Slm1 was sensitive to MIPC levels, Slm1-mNG PM localization would decrease in these strains. We found that Slm1-mNG cortical levels were reduced in *imt1Δ*, *imt2Δ* and *imt3Δ* cells compared to wildtype, and even more reduced in the *imt1Δ imt2Δ imt3Δ* triple deletion compared to any single *imt* deletion (Fig 6E–6F). These data support Slm1 as an indicator of MIPC levels at the intracellular leaflet of the PM.

*S*. *cerevisiae* Slm1 shows reduced PM localization when phosphatidylinositol-4-phosphate (PI4P) and phosphatidylinositol (4,5)-bisphosphate [PI(4,5)P_2_] levels are reduced [86]. Therefore, we next analyzed *S*. *pombe* Slm1-mNG localization in *efr3Δ* cells, which have markedly reduced PI4P and [PI(4,5)P_2_] levels at the PM [87]. Surprisingly, we found that Slm1-mNG septum localization was modestly increased rather than decreased in *efr3Δ* cells compared to wildtype (S5C–S5D Fig). These results suggest that *S*. *pombe* Slm1 localization is a more specific indicator of the level of complex sphingolipids than of PI.

### The PM and glycosylated PM proteins have altered properties in *css1-3* cells

**Fig 7:**
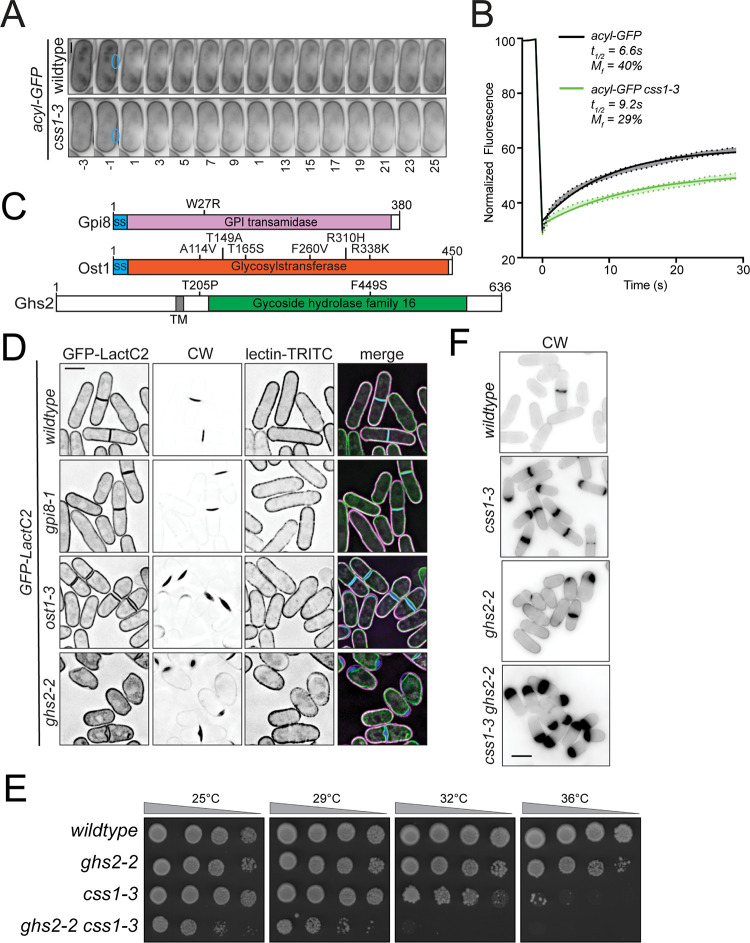
Cells with disrupted glycosylation but not GPI anchor addition phenocopy *css1-3*. (A) Representative live-cell time-lapse movies of wildtype and *css1-3* cells expressing acyl-GFP. Fluorescence of acyl-GFP was photobleached at time 0 and cells were imaged every 1s. Every other time point is shown. Scale bar, 2 μm. (B) Quantification of the fluorescence recovery after photobleaching of the strains from A. Shaded area represent SEM. n = 12. (C) A schematic of Gpi8, Ost1 and Ghs2 with their relevant predicted domains. Mutations in these genes are indicated on the relevant schematic. (D) Live-cell imaging of wildtype, *gpi8-1*, *ost1-3* and *ghs2-2* cells expressing GFP-LactC2. Cells were grown up at 25°C and then shifted to 36°C for 3 h prior to imaging and staining with CW and TRITC-lectin. Scale bar, 5 μm. (E) Serial 10-fold dilutions of the indicated strains were spotted on YE plates and incubated at the indicated temperatures for 3 days. (F) The indicated cells were grown at 25°C and shifted to 36°C for 3 h prior to fixing and staining cells with CW. Scale bar, 5 μm.

The abnormal distribution of sterols and complex sphingolipids along with other bulk changes in the lipid composition led us to ask if the physical properties of the PM are altered in *css1-3* cells. We analyzed the FRAP of acyl-GFP in wildtype and *css1-3* cells as a general indicator of membrane fluidity. We found that acyl-GFP in *css1-3* cells grown at the permissive temperature and then shifted to the restrictive temperature for 1 h had a reduced mobile fraction and a longer half-life than in wildtype cells (Fig 7A–7B) indicating that the PM of *css1-3* cells is more ordered. This finding is consistent with how the concentration of complex sphingolipids affects the organization of the *S*. *cerevisiae* PM [88, 89].

The PM lipid composition, particularly that of sterols and sphingolipids, can influence the conformation of transmembrane domain proteins. This has been demonstrated for human amyloid precursor protein [90–92] and various neurotransmitters [93]. We therefore hypothesized that a PM protein or group of proteins required to incorporate cell wall precursors into the existing cell wall matrix are mis-regulated in *css1-3* and that disrupting these enzymes would phenocopy *css1-3*. We reasoned that such proteins would contain functional extracellular domains and thus be linked to the PM via either a GPI anchor or contain a transmembrane domain. We first disrupted the cellular machinery required for the attachment of GPI anchors by constructing a temperature-sensitive *gpi8* mutant (*gpi8-1*) (Figs 7C and S6A). The *gpi8* gene encodes the ortholog of human PIG-K and *S*. *cerevisiae* Gpi8 that attaches the GPI group onto proteins in the ER [61,94]. We analyzed the phenotype of *gpi8-1* cells expressing the PM marker GFP-lactC2 [95, 96] by also staining with CW and TRITC-lectin. At the restrictive temperature, *gpi8-1* cells did not accumulate cell wall material at their periphery and thus did not phenocopy *css1-3* (Fig 7D). We conclude that the *css1-3* phenotype is unlikely to result from mis-regulation of GPI-anchored proteins.

Because most trans-PM proteins are glycosylated, we next disrupted N-linked glycosylation globally by constructing a temperature sensitive allele of *ost1* (*ost1-3*) (Figs 7C and S6A), which encodes an essential component of the ER-localized oligosaccharyltransferase complex required for this modification [97]. We found that *ost1-3* cells accumulated cell wall deposits at the restrictive temperature, similarly to *css1-3* cells (Fig 7D). These results indicated that disruption of a N-linked glycosylated protein(s), but not a GPI anchored protein(s), may underlie the defect in cell wall construction in *css1-3* cells.

253 proteins are predicted to be N-glycosylated in *S*. *pombe*, of which 240 are N-glycosylated but not GPI-anchored [61]. Of these, 18 are annotated to be involved in cell wall biogenesis and are thus candidates for mis-regulation in *css1-3* cells (S1 Table). Within this list, the uncharacterized protein encoded by SPAC17G6.11c was of particular interest because it is essential, potentially localizes to the PM, and contains a Glycoside Hydrolase 16 domain with homology to laminarinases which are β1,3 glucanases [98]. We named this protein Ghs2 for its Glycoside Hydrolase Sixteen domain 2. We generated a temperature-sensitive *ghs2* allele (*ghs2-2*) (S6B Fig) with defects in phloxine B uptake at high temperatures, a defect also seen with *css1-3* cells. We observed cell wall deposits between the PM and existing cell wall at the restrictive temperature in *ghs2-2* cells (Fig 7D). In support of Ghs2 playing a role in cell wall regulation, negative genetic interactions were observed between *ghs2-2* and *ags1-664*, *bgs1-191* and *bgs4-1* (S6B Fig). *ghs2-2* also displayed a negative genetic interaction with *css1-3* and exacerbated the accumulation of cell wall precursors (Fig 7E and 7F). Thus, we conclude that Ghs2 is a new factor contributing to the coordination of cell wall glucan synthesis and processing.

## Discussion

In this work, we determined the factors responsible for the unique phenotype of the *css1* mutant, in which despite the sustained production and accumulation of cell wall precursors, cell wall extension stops. Our results indicate that an increase in the final product of the sphingolipid synthesis pathway, MIPC, underlies the failure in a final step(s) of cell wall assembly and disrupts the normal coupling of cell growth with construction of the cell wall in *S*. *pombe*. Furthermore, it appears that elevated PM MIPC disrupts the normal intrafacial distribution of PM ergosterols, the properties of the PM, and the functions of N-glycosylated but not GPI-anchored cell wall assembly enzymes that function at the PM. Using a candidate approach, we identified Ghs2 as a previously uncharacterized N-glycosylated PM protein that is key to proper cell wall assembly. Thus, our work has tied global changes in PM composition to the function of specific proteins that couple cell wall construction to cell growth.

The *S*. *cerevisiae* sphingolipid synthesis pathway is well characterized, and most of its enzymes have been conserved throughout evolution [99,100]. Though significantly less studied, the *S*. *pombe* sphingolipid biosynthesis pathway appears similar to that of *S*. *cerevisiae;* orthologs or putative orthologs of pathway enzymes have been identified based on sequence comparisons [44,47,49,61,101]. Also, the final products of the pathway, the complex sphingolipids MIPC in *S*. *pombe* and mannose-(inositol-P)2-ceramide (MIP_2_C) in *S*. *cerevisiae* are not essential for the viability of either yeast species, although defects in morphology, cell wall organization, and stress responses have been reported for mutants defective in their synthesis [47,71,72,102–105]. However, we have revealed a key difference in sphingolipid metabolism between the two yeasts. We found that *S*. *pombe* does not tolerate increases in MIPC, in accord with Css1 being an essential enzyme, and possibly related, forcing the production of MIP_2_C in *S*. *pombe* is also lethal [104]. In contrast, the Css1 ortholog in *S*. *cerevisiae*, Isc1p, is not essential [106]. This significant difference in tolerance for increased complex sphingolipid levels may be explained by a difference in where these lipids accumulate when Css1 or Isc1p are inactivated. *S*. *cerevisiae* Isc1p localizes to the ER [107], suggesting that complex sphingolipids would accumulate there in *isc1∆* cells. However, Css1 is a bona fide PM protein causing an accumulation of complex sphingolipids in the PM.

Another potential explanation for the variable tolerance of the two yeasts to loss of sphingomyelinase-like activities is the difference in overall membrane lipid composition. Our quantitative MS-based lipidomic data provide a similar view of the relative composition of lipid species in *S*. *pombe* as the only other similar study [108]. The one significant difference is the reported abundance of cardiolipin (CL). While we identified a very small amount (~0.4%) of CL, Makarova and colleagues found that ~3% of polar lipid species were CL. Potential reasons for this difference include the use of different yeast growth media and lipid extraction methods. Still, this difference is a small distinction in contrast to the large differences between *S*. *cerevisiae* and *S*. *pombe* lipid composition. Analyses of *S*. *cerevisiae* membrane lipids under a variety of growth conditions has been performed using the same MS-based lipidomic profiling methodology used in this study [109,110]. Although the relative abundance of some lipid species, including IPC and MIPC, are similar between *S*. *cerevisiae* and *S*. *pombe*, there is considerably more PC and far less phosphatidic acid (PA) in *S*. *pombe* (<1% of lipids) relative to *S*. *cerevisiae* (~10% of lipids) [108,110]. Thus, the membranes of *S*. *cerevisiae* might be better able to withstand elevated complex sphingolipid levels than those of *S*. *pombe*.

Css1 is inserted into the PM via two C-terminal transmembrane domains [34], and the catalytic domain is cytosolic. If IPC and MIPC are primarily on the PM’s outer leaflet, it poses a conundrum as to how Css1 is able to hydrolyze them. Based on the dependencies of Slm1 localization (Fig 6), we speculate that IPC and MIPC may be more evenly partitioned between PM leaflets than currently thought. We also propose that the essential role of *S*. *pombe* Css1 might be to prevent MIPC from accumulating to high levels in the PM’s inner leaflet where it might also partition to the outer leaflet, either of which would disrupt PM properties and the functions of PM-resident proteins.

The factors functioning downstream of the biosynthetic enzymes that produce the building blocks of the cell wall are poorly understood in most cell-walled organisms. The *S*. *pombe* genome encodes numerous uncharacterized proteins with putative roles in modifying glucans and galactomannans [61]. We have characterized just one of these, Ghs2, and found that it is key for cell wall assembly downstream of glucan synthases. It is tempting to speculate that as glucan polymers are synthesized and extruded through the PM into the extracellular space, the catalytic domain of Ghs2 would be ideally positioned to process them for incorporation into the existing cell wall matrix. Because of the dramatic changes in PM composition in *css1-3* cells and resulting changes to the physical properties of the PM, the conformations and interactions of such functionally coupled complexes could be grossly disturbed. Our findings may spur further investigation into Ghs2-related proteins that couple with synthetic enzymes to achieve cell wall construction coordinated with cell growth.

## Methods

### Yeast strains, media, and genetic methods

*S*. *pombe* strains used in this study are listed in S2 Table and were grown in yeast extract (YE) media [111]. Crosses were performed on glutamate medium plates and strains were constructed by tetrad analysis. Myriocin (Cayman Chemical Company, cat# 63150) was used at 400 ng/mL and aureobasidin A (Takara, cat# 630466) was used at 20 ng/mL added to YE media and agar plates.

*mtl2*, *bgs1*, *css1*, *css1-3*, *slm1*, *ppk14* and *dnf1* were tagged endogenously at the 3′ end of their open reading frames (ORFs) with mNeonGreen:kanMX6 (mNG), mNG:hphMX6 or moxNG (mNG with C149S):kanMX6 using pFA6 cassettes as previously described [83,112,113]. moxNG, an oxidizing environment-optimized variant of mNG was constructed by mutating cystine 149 to serine in mNG [83].

A lithium acetate transformation method was used for introducing sequences encoding tags, and integration of tags was verified using whole-cell PCR and/or microscopy. G418 (100 mg/mL; Sigma-Aldrich, #11811031) and Hygromycin B (50 mg/mL; Thermo Fisher, #10687010) in YE media was used for selecting *kan*^*R*^ or *hyg*^*R*^ cells, respectively. Introduction of tagged loci into other genetic backgrounds was accomplished using standard *S*. *pombe* mating, sporulation, and tetrad dissection techniques. Fusion proteins were expressed from their native promoters at their normal chromosomal locus unless otherwise indicated. Expression of *acyl-GFP*, integrated into the *leu1* locus [114], was controlled by the thiamine-repressible *nmt1* promoter. Expression from the *nmt1* promoter was repressed by addition of 5 μg/mL thiamine to the medium, and expression was induced by washing and culturing in medium lacking thiamine for at least 24 h at 25°C. For serial dilution growth assays, cells were cultured in liquid YE at 25°C, three or four serial 10-fold dilutions starting at 4 × 10^6^ cells/mL were made, 3 μL of each dilution was spotted on YE plates, and cells were grown at the indicated temperatures for two days.

### PCR mediated gene deletions

*erg4*, *erg5*, *dnf2*, *dnf2*.*5* gene deletions were made as previously described [115]. *imt3* and *dnf1* gene deletions were obtained using a two-step PCR, except that the open reading frames (ORFs) were replaced with *ura4*^*+*^ rather than *kan*MX6. Transformants were selected on plates lacking uracil at 29°C for 3–5 days and colonies were checked by three PCR reactions to ensure loss of the ORF and correct insertion of *ura4*^*+*^ as previously described [115].

### Isolation of cold-sensitive *cut6-1* mutant

Suppressor mutations of *css1-3* at 36°C were isolated by nitrosoguanidine mutagenesis as described [111]. These mutants were then crossed to *css1-3* twice to determine if the suppressor mutation segregated as a single extragenic locus. Those that did were then tested for cold-sensitive growth at 19°C. Two cold-sensitive double mutants were isolated, outcrossed to wildtype three times, and the suppressor mutants were found to be cold-sensitive on its own. One was crossed to wildtype a fourth time and from those tetrads dissections, DNA was extracted from 8 wildtype and 8 mutant colonies of the same four tetrads. Equal amounts of genomic DNA from wildtype and mutant colonies were sequenced and a single base pair difference was identified between them at 100% frequency that led to a T to C mutation at position 6351 of the *cut6* open reading frame. This in turn led to a substitution of residue L1910 to serine within the Cut6 acetyl-coenzymeA carboxyl transferase domain. This mutation was confirmed to exist in the original extragenic mutation and all cold-sensitive strains arising from it. We named this *cut6* cold-sensitive mutant *cut6-1*. DNA sequencing of *cut6* from the second cold-sensitive suppressor mutant led to the identification of a different missense mutation that changes K1528 to R.

### Isolation of temperature sensitive alleles with error-prone PCR

Temperature sensitive alleles of *gpi8*, *ost1* and *ghs2* were constructed based on the previously described protocol [116] but used EX taq polymerase (Takara, 4025) and accompanying dNTPs (Takara, RR01BM).

### Microscopy methods

All live-cell images of *S*. *pombe* cells were acquired using a Personal DeltaVision microscope system (Leica Biosystems), which includes an Olympus IX71 microscope, 60×NA 1.42 Plan Apochromat and 100×NA 1.40 U Plan S Apochromat objectives, live-cell and standard filter wheel sets, softWoRx imaging software, and either a Photometrics CoolSnap HQ2 camera or a pco.edge 4.2 sCMOS camera. z-sections spaced at 0.2–0.5 μm. Images were deconvolved with 10 iterations.

For live-cell time-lapse imaging, cells were secured within a CellASIC ONIX microfluidics perfusion system (Millipore Sigma). Cells were loaded into Y04C plates for 5 sec at 8 psi, and YE liquid media flowed into the chamber at 5 psi during imaging. Single z-planes were imaged every 20 min.

Fluorescence intensity measurements were made with ImageJ software (National Institutes of Health) [117]. All intensity measurements were corrected for background. In each image used, background intensity measurements were taken from an area without any cells, which was divided by that area to give the average intensity per pixel of the background. This value was then multiplied by the area of the region of interest (ROI) and subtracted from that ROI’s raw intensity measurement to get the intensity measurement corrected for background. For whole cell fluorescence measurements, the corrected intensity measurements were divided by the area of the ROI.

To account for autofluorescence at the 3-hour time point in S4A–S4C Fig, cells lacking fluorescent tags but otherwise isogenic backgrounds were imaged and the fluorescence intensity was quantified from summed whole cell *Z*-projections (n = 30). The autofluorescence measurement was normalized to the wildtype strain and applied to the whole cell fluorescence intensity. For cell tip intensity measurements, a 44 pixel (4.71 μm) diameter circle ROI was applied to cell tips to acquire an intensity measurement. For septum intensity measurements, an ROI was drawn around the septum.

For CW staining, 50 μL of a 5 μg/mL stock of CW in PBS was added to 1 mL of cells 5 min before imaging. For TRITC-Lectin labeling, 1 μL of a 5 mg/mL stock of TRITC-lectin in water was added to 1 mL of cells for a final concentration of 5 μg/mL. Cells were then nutated for 10 min at room temperature, washed three times, and resuspended in PBS. For filipin staining the drug was added at final concentration of 10 μg/mL from the DMSO stock to the cells. Cells were imaged live within 5 min.

For all line scans, a medial slice of non-deconvolved images were used. For line scans in Fig 3D, intensity measurements were plotted against distance across the short cell axis on the cells shown in Fig 3B. For line scans in Figs 4F and 5C, intensity measurements of 5 cells with a septum were taken across the middle of the long cell axis per genotype or condition (as depicted in the schematic Fig 4F, right and Fig 5C, top) and plotted against distance. For line scans in Fig 6D, intensity measurements of 10 cells were plotted against distance across the short cell axis per genotype.

All images used for quantification were not deconvolved and were sum projected. Images in Figs 1, 3–7, and S3 and S5C are deconvolved medial slices. Images in S1 and S5B Figs are deconvolved max projected images.

FRAP analysis was performed on the Personal DeltaVision microscope described above. An acyl-GFP membrane spot was photobleached with a 488 laser. Images were acquired every 1s for 30s and three images were taken prior to the photobleaching event. Quantifications of the fluorescence recovery were performed using ImageJ. FRAP intensity measurements were corrected for background and time-course photobleaching. Each FRAP measurement was obtained from a separate cell, and a minimum of 12 cells were analyzed in three separate experiments.

### Quantification and statistical analysis

Calculations of standard error of the mean (SEM), and statistical significances were performed with Prism 8.4.2 (GraphPad Software). Significance was defined by a p value equal to or less than 0.05. For all data following a normal distribution, a Student’s two-sample unpaired t test or an ANOVA test was used with Tukey’s post hoc analysis. Sample sizes and the numbers of replications are included in the Fig graphs or legends. Where indicated, n represents the number of cells used for quantification. For all image analyses, no raw data were excluded with the exception of cells that were not in focus or if a cell moved during imaging.

### Lipidomic analysis

3 x 10^8^ cells were cultured in liquid YE at 25°C and then shifted to 36°C for 1 h. After shift, cells were collected by centrifugation, washed three times with PBS, and then lysed with glass beads in 1 mL of water using a Fast Prep device (MP Biomedical). Lipids were extracted using chloroform and methanol. Samples were spiked with lipid class-specific internal standards prior to extraction. After drying and resuspending in MS acquisition mixture, lipid extracts were subjected to mass spectrometric analysis. Mass spectra were acquired on a hybrid quadrupole/Orbitrap mass spectrometer equipped with an automated nano-flow electrospray ion source in both positive and negative ion mode.

Lipid identification using LipotypeXplorer was performed on unprocessed (*.raw format) mass spectra. For MS-only mode, lipid identification was based on the molecular masses of the intact molecules. MSMS mode included the collision induced fragmentation of lipid molecules and lipid identification was based on both the intact masses and the masses of the fragments. Prior to normalization and further statistical analysis lipid identifications were filtered according to mass accuracy, occupation threshold, noise and background. Lists of identified lipids and their intensities were stored in a database optimized for the particular structure inherent to lipidomic datasets. Intensity of lipid class-specific internal standards was used for lipid quantification.

The dynamic range for yeast total cell lysate samples was determined prior to analysis. Based on these data, limits of quantification and coefficients of variation for the different lipid classes were determined. Limits of quantification are in the lower μM to sub-μM range, depending on the lipid class. The average coefficient of variation for a complete set of quantified lipid classes is around 10–15%.

Each analysis is accompanied by a set of blank samples to control for a background and a set of quality control reference samples to control for intra-run reproducibility and sample specific issues.

## Supporting information

S1 Fig Glucan synthases contribute to the *css1-3* phenotype. (A) Live-cell imaging of endogenously tagged *ags1-RFP*, *bgs1-mNG*, *GFP-bgs3* and *GFP-bgs4* in wildtype and *css1-3* cells at 25°C and after shifting to 36°C for 3 h. Scale bar, 5 μm. (B) Serial 10-fold dilutions of the indicated strains were spotted on YE plates and incubated at the indicated temperatures. (C) The indicated strains were grown at 25°C in YE or YE containing 1.2 M sorbitol and then shifted to 36°C for 3 h prior to fixing and staining with CW. Scale bar, 5 μm.
(PDF)

S2 Fig Mutations in the Cell Integrity Pathway suppress *css1-3*. (A) Serial 10-fold dilutions of the indicated strains were spotted on YE plates and incubated at the indicated temperatures. (B) Live-cell imaging of *wildtype* or *css1-3* cells expressing *wsc1-mNG* and grown at 25°C and shifted to 36°C for 3 h prior to imaging. Cells were stained with TRITC-lectin. Scale bar, 5 μm.
(PDF)

S3 Fig Mutations in the sphingolipid biosynthesis pathway suppress *css1-3*. (A-D) Serial 10-fold dilutions of the indicated strains were spotted on YE plates and incubated at the indicated temperatures. Myriocin was used at 400 ng/ml and aureobasidin A was used at 20 ng/ml. (E) The indicated strains grown at 25°C and shifted to 36°C for 3 h prior to fixing and staining with CW. (F) Live-cell imaging of cells grown up at 25°C and shifted to 36°C for 3 h prior to imaging. Cells were stained with CW and TRITC-lectin. Scale bar, 5 μm.
(PDF)

S4 Fig Mutations in the sterol biosynthesis pathway suppress *css1-3*. (A) Live-cell imaging of wildtype and *css1-3* cells expressing sterol sensor *mCherry-D4H* and grown at 25°C then shifted to 36°C for 3 h and imaged each h. (B) Quantification of fluorescence intensity per μm^2^ of whole non-septated cells from A. n = 45. (C) Quantification of septum intensity of cells from A. n ≥ 35. (D) Quantification of cell tip intensity from A. n ≥ 54. For B, C and D error bars represent SEM. ****p ≤ 0.0001; one-way ANOVA. In B, *css1-3* 25°C vs *css1-3* 3h 36°C, p = 0.096 (E) Fixed-cell imaging of the wildtype and *css1-3* cells stained with filipin prior to imaging. (F) Serial 10-fold dilutions of the indicated strains were spotted on YE plates and incubated at the indicated temperatures. (G) The indicated strains grown at 25°C and shifted to 36°C for 3 h prior to fixing and staining with CW. Scale bars, 5 μm.
(PDF)

S5 Fig Slm1 localization is not altered in *efr3Δ*. (A) Serial 10-fold dilutions of the indicated strains were spotted on YE plates and incubated at the indicated temperatures. (B) Live-cell imaging of *ppk14-mNG* and *dnf1-moxNG* expressing cells grown at 25°C. (C) Live-cell imaging of wildtype and *efr3Δ* cells expressing Slm1-mNG grown up at 25°C. (D) Quantification of Slm1-mNG septum intensity from A. n = 45. ****p ≤ 0.0001; Student’s t-test. Scale bars, 5 μm.
(PDF)

S6 Fig Characterization of *gpi8-1*, *ost1-3* and *ghs2-2* temperature sensitive alleles. A-B) Serial 10-fold dilutions of the indicated strains were spotted on YE plates and incubated at the indicated temperatures.
(PDF)

S1 Table List of proteins of interest that may be misregulated in *css1-3*. (PDF)

S2 Table List of fission yeast strain used in this study. (PDF)
