# Supplementary figures and images for: Elevated levels of sphingolipid MIPC in the plasma membrane disrupt the coordination of cell growth with cell wall formation in fission yeast
Source: PLoS Genet. 2023 Oct 4;19(10):e1010987. doi: 10.1371/journal.pgen.1010987 (PMC10578601; doi:10.1371/journal.pgen.1010987)

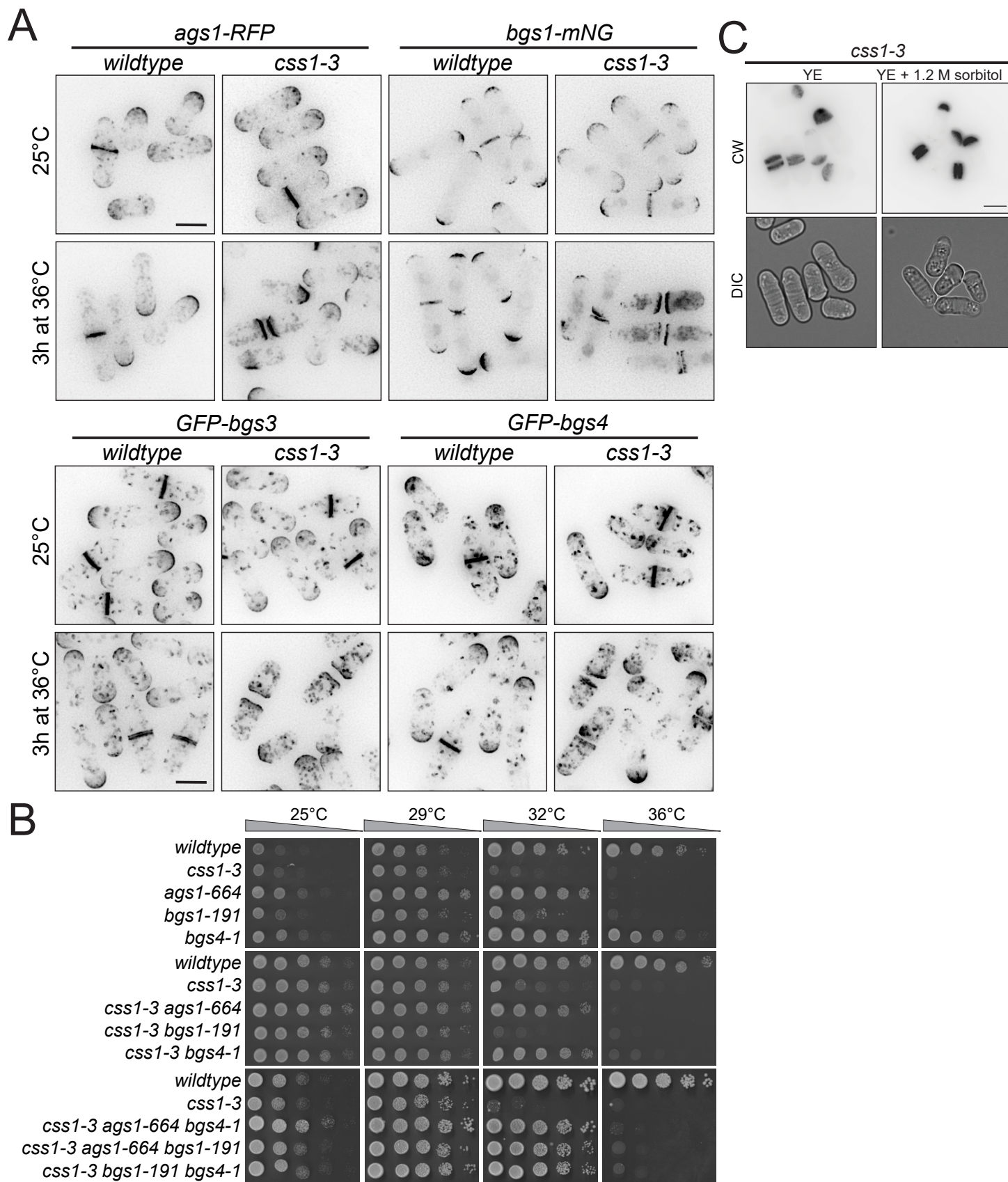

Supplement: S1 Fig — (A) Live-cell imaging of endogenously tagged ags1-RFP, bgs1-mNG, GFP-bgs3 and GFP-bgs4 in wildtype and css1-3 cells at 25°C and after shifting to 36°C for 3 h. Scale bar, 5 μm. (B) Serial 10-fold dilutions of the indicated strains were spotted on YE plates and incubated at the indicated temperatures. (C) The indicated strains were grown at 25°C in YE or YE containing 1.2 M sorbitol and then shifted to 36°C for 3 h prior to fixing and staining with CW. Scale bar, 5 μm. (PDF) [file pgen.1010987.s001.pdf]

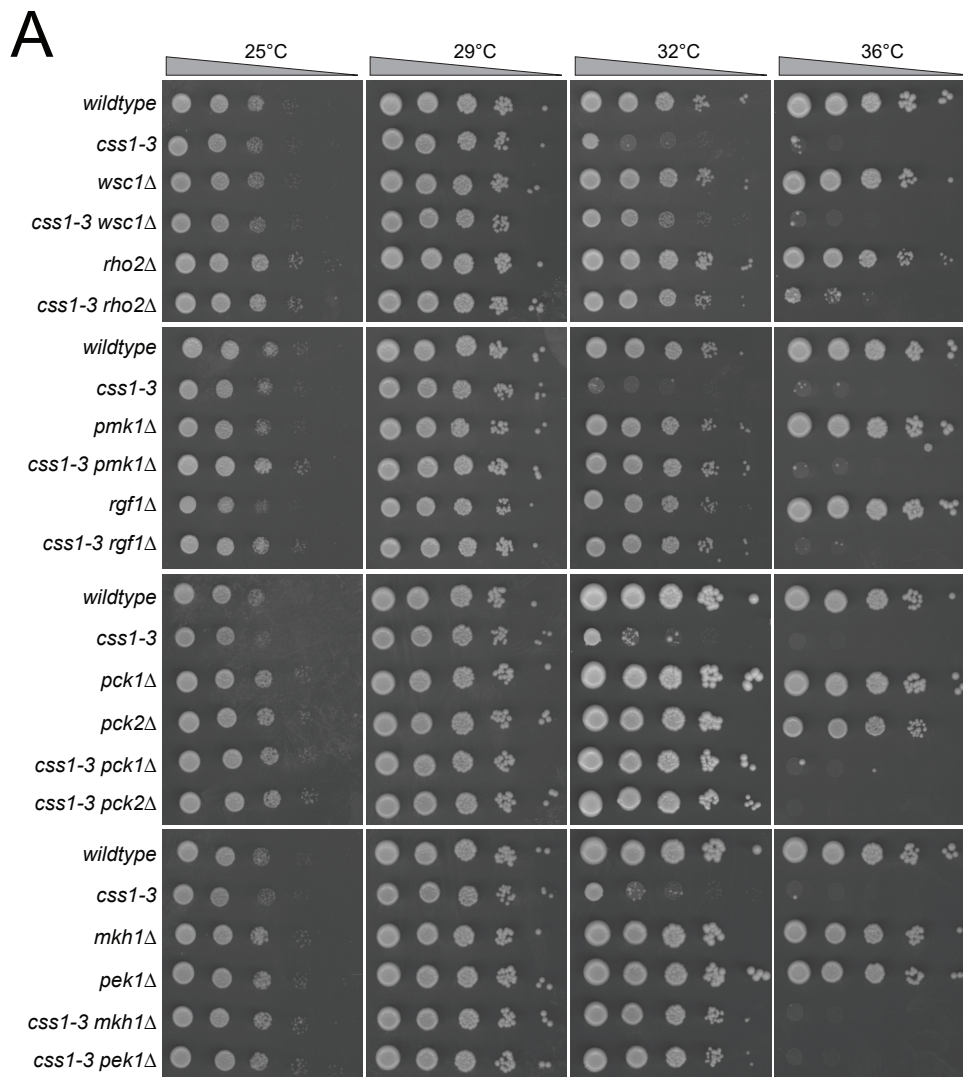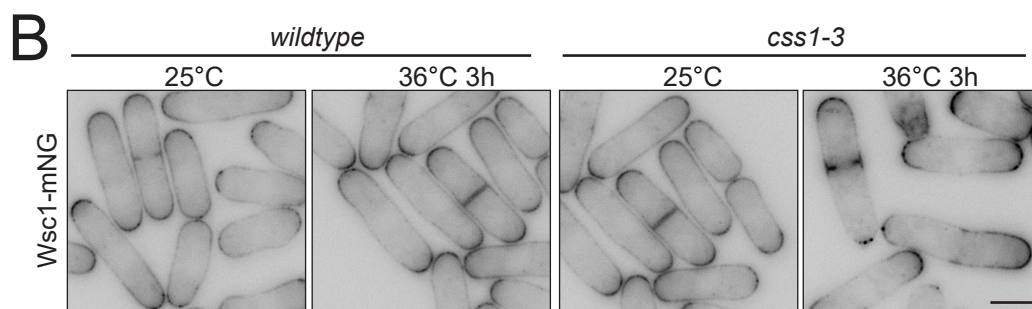

Supplement: S2 Fig — (A) Serial 10-fold dilutions of the indicated strains were spotted on YE plates and incubated at the indicated temperatures. (B) Live-cell imaging of wildtype or css1-3 cells expressing wsc1-mNG and grown at 25°C and shifted to 36°C for 3 h prior to imaging. Cells were stained with TRITC-lectin. Scale bar, 5 μm. (PDF) [file pgen.1010987.s002.pdf]

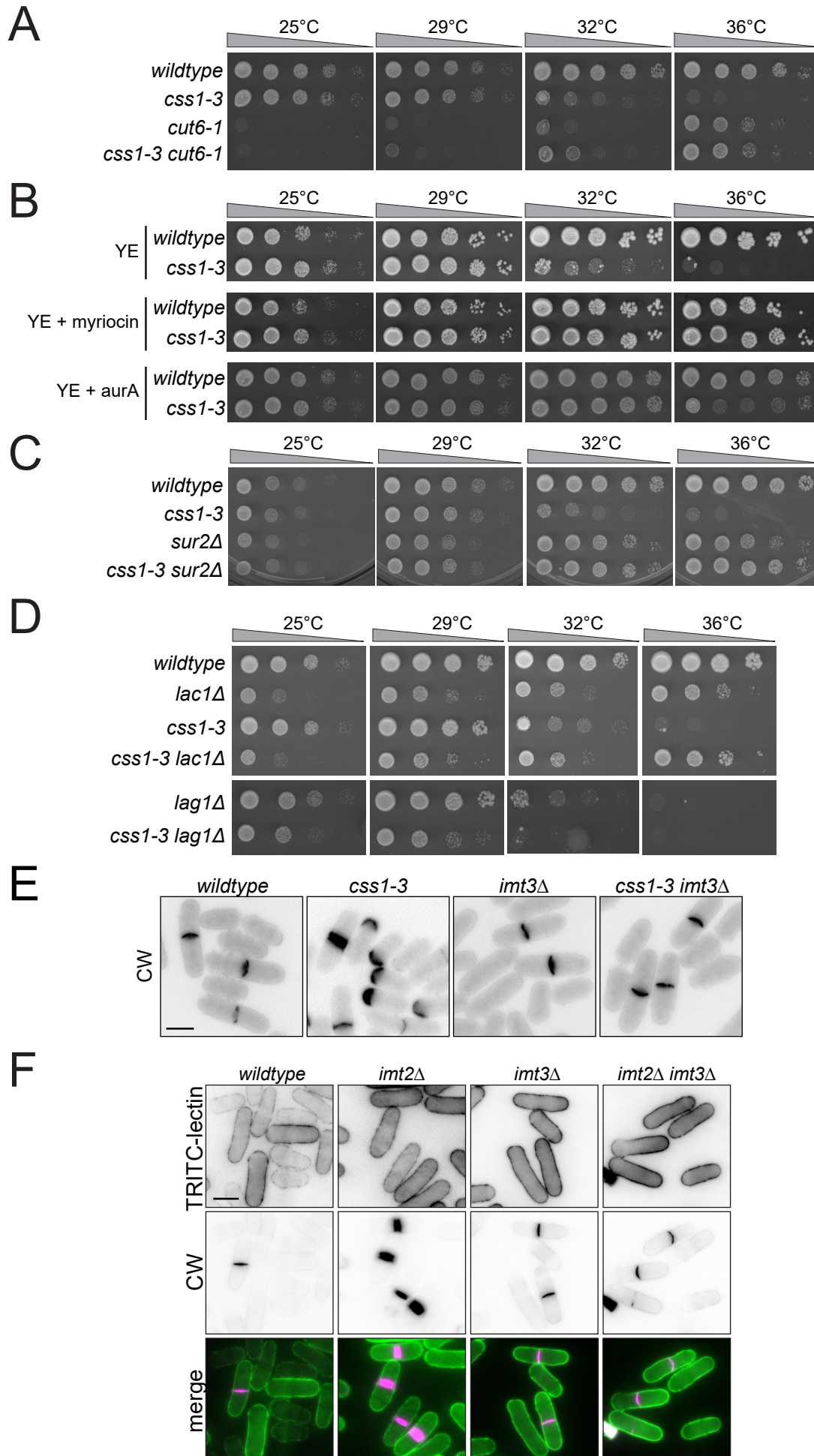

Supplement: S3 Fig — (A-D) Serial 10-fold dilutions of the indicated strains were spotted on YE plates and incubated at the indicated temperatures. Myriocin was used at 400 ng/ml and aureobasidin A was used at 20 ng/ml. (E) The indicated strains grown at 25°C and shifted to 36°C for 3 h prior to fixing and staining with CW. (F) Live-cell imaging of cells grown up at 25°C and shifted to 36°C for 3 h prior to imaging. Cells were stained with CW and TRITC-lectin. Scale bar, 5 μm. (PDF) [file pgen.1010987.s003.pdf]

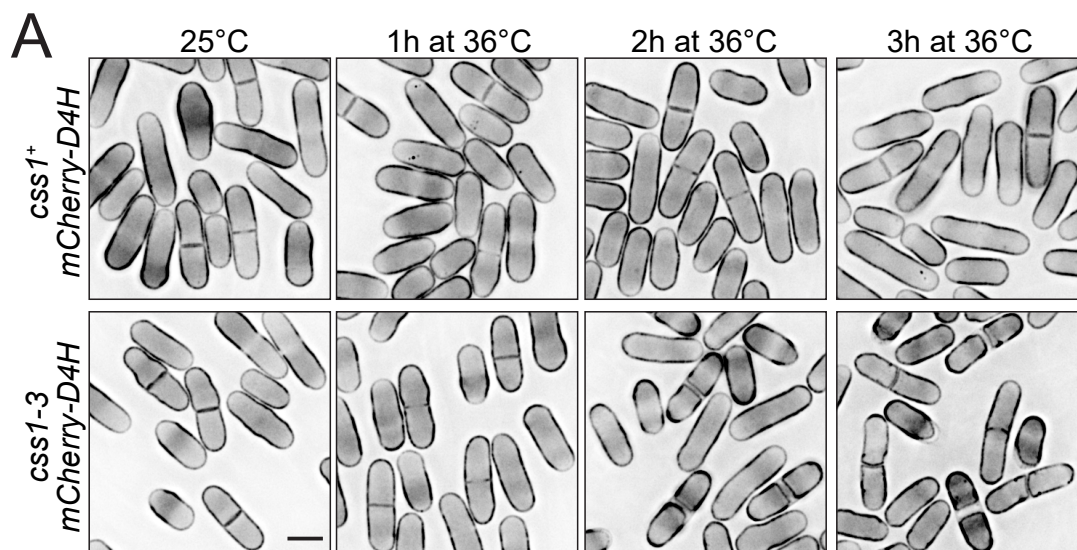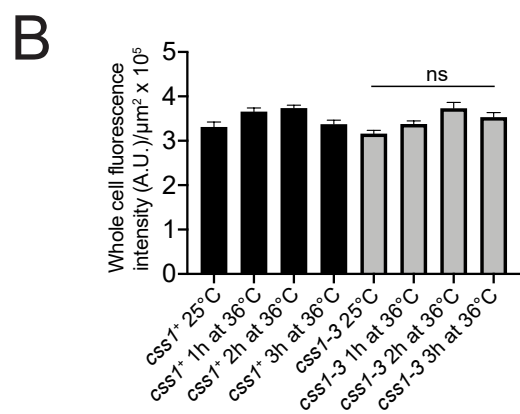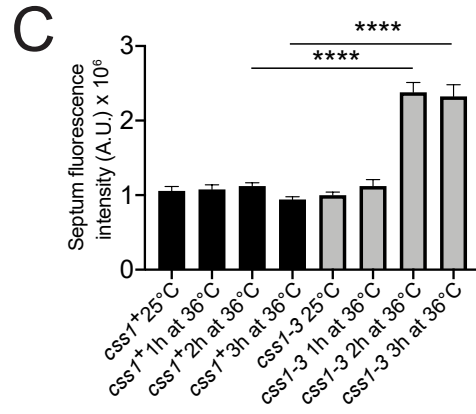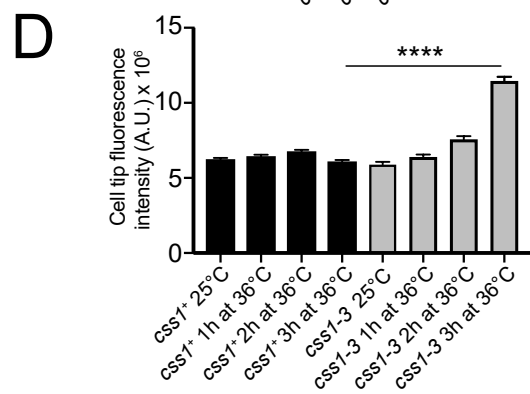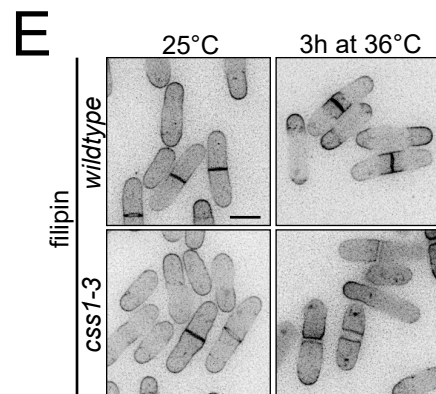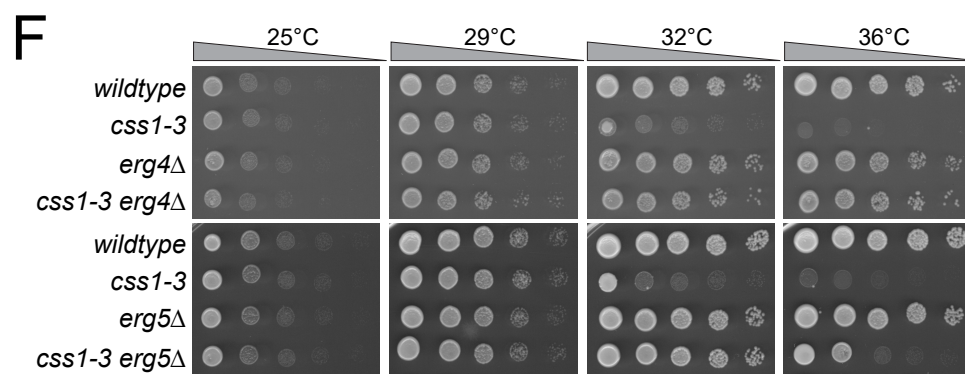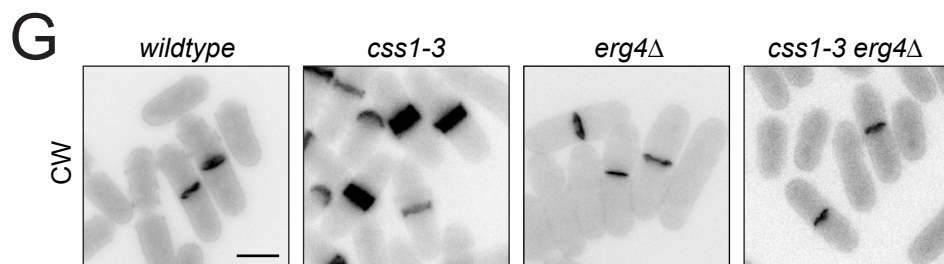

Supplement: S4 Fig — (A) Live-cell imaging of wildtype and css1-3 cells expressing sterol sensor mCherry-D4H and grown at 25°C then shifted to 36°C for 3 h and imaged each h. (B) Quantification of fluorescence intensity per μm2 of whole non-septated cells from A. n = 45. (C) Quantification of septum intensity of cells from A. n ≥ 35. (D) Quantification of cell tip intensity from A. n ≥ 54. For B, C and D error bars represent SEM. ****p ≤ 0.0001; one-way ANOVA. In B, css1-3 25°C vs css1-3 3h 36°C, p = 0.096 (E) Fixed-cell imaging of the wildtype and css1-3 cells stained with filipin prior to imaging. (F) Serial 10-fold dilutions of the indicated strains were spotted on YE plates and incubated at the indicated temperatures. (G) The indicated strains grown at 25°C and shifted to 36°C for 3 h prior to fixing and staining with CW. Scale bars, 5 μm. (PDF) [file pgen.1010987.s004.pdf]

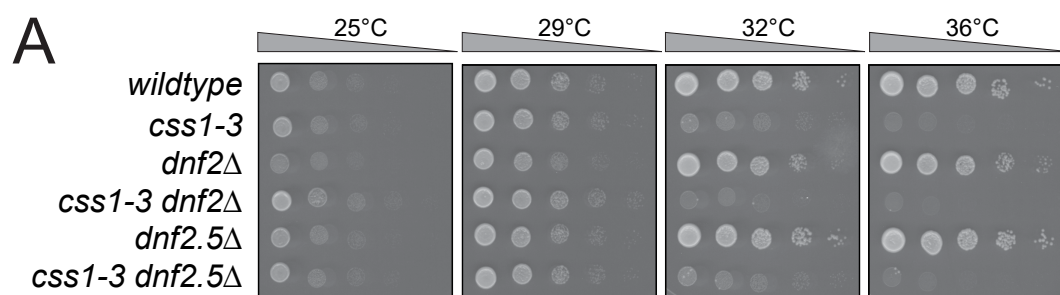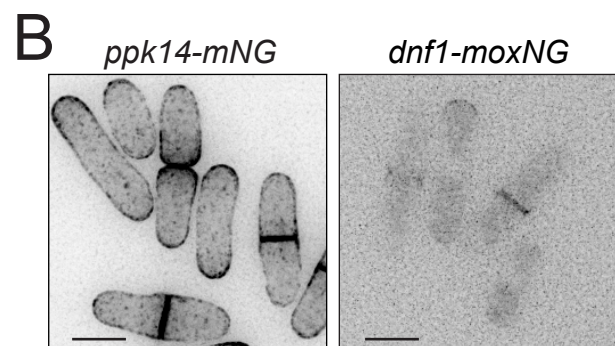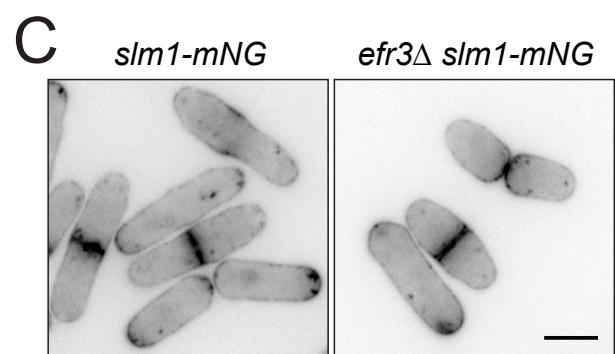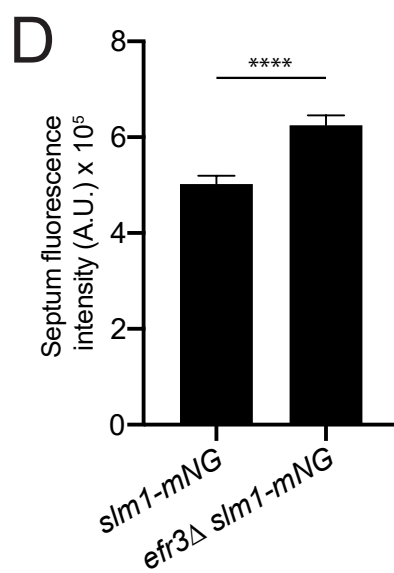

Supplement: S5 Fig — (A) Serial 10-fold dilutions of the indicated strains were spotted on YE plates and incubated at the indicated temperatures. (B) Live-cell imaging of ppk14-mNG and dnf1-moxNG expressing cells grown at 25°C. (C) Live-cell imaging of wildtype and efr3Δ cells expressing Slm1-mNG grown up at 25°C. (D) Quantification of Slm1-mNG septum intensity from A. n = 45. ****p ≤ 0.0001; Student’s t-test. Scale bars, 5 μm. (PDF) [file pgen.1010987.s005.pdf]

A

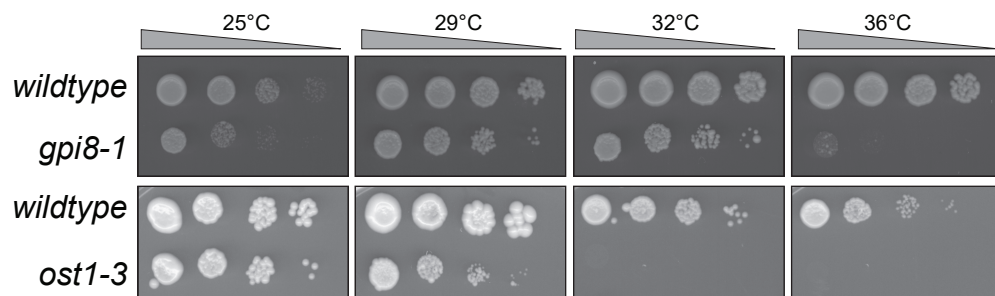

B

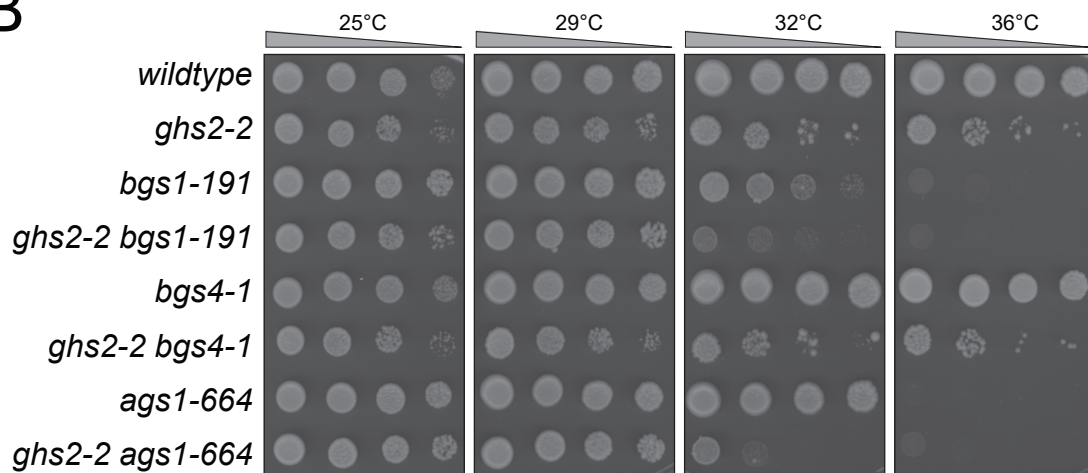

Supplement: S6 Fig — A-B) Serial 10-fold dilutions of the indicated strains were spotted on YE plates and incubated at the indicated temperatures. (PDF) [file pgen.1010987.s006.pdf]
