# Supplementary material for: Elevated levels of sphingolipid MIPC in the plasma membrane disrupt the coordination of cell growth with cell wall formation in fission yeast
Source: PLoS Genet. 2023 Oct 4;19(10):e1010987. doi: 10.1371/journal.pgen.1010987 (PMC10578601; doi:10.1371/journal.pgen.1010987)
Supplement: S1 Table — (PDF) [file pgen.1010987.s007.pdf]

S1 TABLE *Protein list of candidates misregulated in css1-3*

Systematic ID	Gene name	Product description	Essential?	At the PM during vegetative growth?
SPAC637.06	gmh5	alpha-1,2-galactosyltransferase	no	no
SPBC19C7.12c	omh1	alpha-1,2-mannosyltransferase Omh1	no	no
SPBC16H5.09c	omh2	alpha-1,2-mannosyltransferase Omh2	no	no
SPBC32H8.08c	omh5	alpha-1,2-mannosyltransferase Omh5	no	no
SPBC16D10.05	mok13	alpha-1,3-glucan synthase Mok13	no	no
SPCC1281.01	ags1	cell wall alpha-1,3-glucan synthase Ags1	Yes	Yes
SPCC1840.02c	bgs4	cell wall and secondary septum 1,6 branched 1,3-beta-glucan synthase catalytic subunit Bgs4	Depends	Yes
SPBC1105.05	exg1	cell wall glucan 1,6-beta-glucosidase Exg1	no	Yes
SPAPB1E7.04c	cts2	chitinase Cts2	no	Yes
SPAC26H5.08c	bgl2	glucan beta-glucosidase Bgl2	no	Yes
SPBC21B10.07	crr1	glycosyl hydrolase family Crr1	no	Yes
SPAC27E2.07	pvg2	Golgi 4,6-pyruvylated galactose (PvGal) residue biosynthesis protein Pvg2	no	no
SPAC8F11.10c	pvg1	Golgi pyruvyltransferase Pvg1	no	no
SPBC1198.07c	dfg502	mannan endo-1,6-alpha-mannosidase Dfg502	no	no
SPAC22A12.07c	ogm1	protein O-mannosyltransferase Ogm1	no	no
SPBC16C6.09	ogm4	protein O-mannosyltransferase Ogm4	no	no
SPCC645.07	rgf1	RhoGEF for Rho1, Rgf1	no	Yes
SPAC17G6.11c	ghs2	sphingolipid biosynthesis protein	Yes	Yes
