# Supplementary material for: Elevated levels of sphingolipid MIPC in the plasma membrane disrupt the coordination of cell growth with cell wall formation in fission yeast
Source: PLoS Genet. 2023 Oct 4;19(10):e1010987. doi: 10.1371/journal.pgen.1010987 (PMC10578601; doi:10.1371/journal.pgen.1010987)
Supplement: S2 Table — (PDF) [file pgen.1010987.s008.pdf]

S2 Table *S. pombe* strains used in this study

Strain	Genotype	Source
**Figure 1**		
KGY246	*ade6-M210 leu1-32 ura4-D18* h^-	Lab stock
KGY1171	*css1-3 ade6-M210 leu1-32 ura4-D18* h^-	Lab stock
KGY9505-2	*css1-3 leu1:acyl-GFP ura4-D18 ade6-M210* h^-	This study
KGY16378	*leu1:acyl-GFP ura4-D18 ade6-M210* h^-	Lab stock
KGY1095-2	*css1-3 cut11-GFP:*$ura4^+$ *ade6-M210 leu1-32 ura4-D18* h^-	This study
KGY19806	*mtl2-mNG:kanMX6 ade6-M210 ura4-D18 leu1-32* h^-	This study
KGY19816	*css1-3 mtl2-mNG:kanMX6 ade6-M210 ura4-D18 leu1-32* h^+	This study
**Figure 2**		
KGY15769	*imt1Δ::kanMX6 ade6-M210 ura4-D18 leu1-32* h^+	Bioneer V3
KGY3442-2	*imt2Δ::kan ade6-M210 ura4-D18 leu1-32* h^+	Bioneer V3
KGY3443-2	*imt3Δ::*$ura4^+$ *ade6-M210 ura4-D18 leu1-32* h^+	This study
KGY5741-2	*imt1Δ::kanMX6 imt2ΔΔ::kanMX6 ade6-M210 ura4-D18 leu1-32* h^-	This study
KGY5495-2	*imt1Δ::kanMX6 imt3Δ::*$ura4^+$ *ade6-M210 ura4-D18 leu1-32* h^+	This study
KGY5655-2	*imt2Δ::kanMX6 imt3Δ::*$ura4^+$ *ade6-M210 ura4-D18 leu1-32* h^+	This study
KGY5778-2	*imt1Δ::kanMX6 imt2Δ::kanMX6 imt3Δ::ura4 ade6-M210 leu1-32* h^+	This study
KGY3444-2	*css1-3 imt1Δ::kanMX6 ade6-M210 ura4-D18 leu1-32,* h^+	This study
KGY3450-2	*css1-3 imt2Δ::kanMX6 ade6-M210 ura4-D18 leu1-32* h^-	This study
KGY3451-2	*css1-3 imt3Δ::*$ura4^+$ *ade6-M210 leu1-32* h^+	This study
KGY6185-2	*css1-3 imt1Δ::kanMX6 imt2Δ::kanMX6 ade6-M210 ura4-D18 leu1-32* h^+	This study
KGY6152-2	*css1-3 imt1Δ::kanMX6 imt3Δ::*$ura4^+$ *ade6-M210 ura4-D18 leu1-32* h^+	This study
KGY5908-2	*css1-3 imt2Δ::kanMX6 imt3Δ::*$ura4^+$ *ade6-M210 ura4-D18 leu1-32* h^+	This study
KGY6134-2	*css1-3 imt1Δ::kanMX6 imt2Δ::kanMX6 imt3Δ::*$ura4^+$*ade6-M210 leu1-32* h^-	This study
**Figure 3**		
KGY2640-2	*css1-mNG:kanMX6* $leu1^+$*:pbip1-mCherry-AHDL ura4-D18 ade6-M210* h^+	This study
KGY1968-2	*css1-mNG:kanMX6* $leu1^+$*:pbip1-mCherry-AHDL scs2Δ::*$ura4^+$ *scs22Δ::*$ura4^+$ *ura4-D18 ade6-M210* h^-	This study
KGY1590-2	*css1-mNG:kanMX6 ura4-D18 leu1-32 ade6-M210* h^-	This study
KGY19834	*css1-3-mNG:kanMX6 ura4-D18 leu1-32 ade6-M210* h^+	This study
KGY7851-2	*css1-GBP-mCherry:kanMX6 ura4-D18 leu1-32 ade6-M210* h^+	This study
KGY19515	*ank1-GFP:kanMX6 ura4-D18 leu1-32 ade6-M210* h^-	This study
KGY7858-2	*css1-GBP-mCherry:kanMX6 ank1-GFP:kanMX6 ura4-D18 leu1-32 ade6-M210* h^+	This study
**Figure 4**		
KGY19756	$ura4^+$*:Pact1-sfGFP-D4H ade6-M210 ura4-D18 leu1-32* h^{90}	[1]

KGY19757	*css1-3 ura4^{+}:Pact1-sfGFP-D4H ade6-M210 ura4-D18 leu1-32 h^{-}*	This study
**Figure 5**		
KGY6983	*ppk14Δ::ura4^{+} ade6-M210 ura4-D18 leu1-32 h^{+}*	[2]
KGY3564-2	*ppk14Δ::ura4^{+} css1-3 ade6-M210 ura4-D18 leu1-32 h^{+}*	This study
KGY3917-2	*dnf1Δ::ura4^{+} ade6-M210 ura4-D18 leu1-32 h^{+}*	This study
KGY3918-2	*dnf1Δ::ura4^{+} css1-3 ade6-M210 ura4-D18 leu1-32 h^{-}*	This study
KGY4618-2	*ura4^{+}Δ::pACT1-sfGFP-D4H dnf1Δ::ura4^{+} ade6-M210 leu1-32 ura4-D18 h^{-}*	This study
**Figure 6**		
KGY19005	*slm1-mNG:kanMX6 ura4-D18 ade6-M210 leu1-32 h^{-}*	This study
KGY955-2	*slm1-mNG:kanMX6 css1-3 ura4-D18 ade6-M210 leu1-32 h^{-}*	This study
KGY1281-2	*slm1-mNG:kanMX6 imt3Δ::ura4^{+} ura4-D18 ade6-M210 leu1-32 h^{-}*	This study
KGY5475-2	*slm1-mNG:kanMX6 css1-3 imt3Δ::ura4^{+} ura4-D18 ade6-M210 leu1-32 h^{-}*	This study
KGY5711-2	*slm1-mNG:kanMX6 imt1Δ::kanMX6 ura4-D18 ade6-M210 leu1-32 h^{+}*	This study
KGY5642-2	*slm1-mNG:kanMX6 imt2Δ::kanMX6 ura4-D18 ade6-M210 leu1-32 h^{-}*	This study
KGY5760-2	*slm1-mNG:kanMX6 imt1Δ::kanMX6 imt2Δ::kanMX6 imt3Δ::ura4 ura4-D18 ade6-M210 leu1-32 h-*	This study
**Figure 7**		
KGY16378	*Pnmt1-acyl-GFP:leu1^{+} ura4-D18 ade6-M210 h^{-}*	Lab stock
KGY2997-3	*Pact1-lactC2-GFP:natMX6 ura4-D18 ade6-M210 leu1-32 h^{-}*	[3]
KGY5772-2	*Pact1-lactC2-GFP:natMX6 gpi8-1:kanMX6 ura4-D18 ade6-M210 leu1-32 h^{-}*	This study
KGY4901-2	*Pact1-lactC2-GFP:natMX6 ost1-3:kanMX6 ura4-D18 ade6-M210 leu1-32 h-*	This study
KGY6294-2	*Pact1-lactC2-GFP:natMX6 ghs2-2:kanMX6 ura4-D18 ade6-M210 leu1-32 h^{-}*	This study
KGY6301-2	*ghs2-2:kanMX6 ade6-M210 ura4-D18 leu1-32 h^{-}*	This study
KGY7022-2	*css1-3 ghs2-2:kanMX6 ade6-M210 ura4-D18 leu1-32 h^{-}*	This study
**S1 Figure**		
KGY2440	*mok1-664 (ags1-664) leu1-32 h^{-}*	[4]
KGY2153	*cps1-191 (bgs1-191) ura4-D18 ade6-M210 lys1-131 h^{-}*	Lab stock
KGY11106	*cwg1-1 (bgs4-1) leu1-32 h^{-}*	[5]
KGY2637-2	*css1-3 mok1-664 cpc1-191 ura4-D18 ade6-M210 lys1-131 h^{90}*	This study
KGY2639-2	*css1-3 cpc1-191 cwg1-1 ura4-D18 ade6-M210 lys1-131 h^{+}*	This study
KGY2638-2	*css1-3 mok1-664 cwg1-1 ura4-D18 leu1-32 ade6-M21X h^{-}*	This study
KGY2651-2	*css1-3 ags1Δ::ura4^{+} ags1-RFP:leu1^{+} ade6-M210 h^{+}*	This study
KGY2652-2	*css1-3 bgs1-mNG:hphMX6 ura4-D18 leu1-32 ade6-M210 h-*	This study
KGY2653-2	*css1-3 bgs4Δ::ura4^{+} GFP-bgs4:leu1^{+} ade6-M210 h^{+}*	This study
KGY2701-2	*css1-3 bgs3Δ::ura4^{+} GFP-bgs3:leu1^{+} ade6-M210 h^{+}*	This study
KGY1937-2	*ags1Δ::ura4^{+} ags1-RFP:leu1^{+} ade6-M210 h^{+}*	[6]

KGY509-2	*bgs1-mNG:hphMX6 ura4-D18 leu1-32 ade6-M210 h*$^{-}$	This study
KGY11110	*bgs4Δ::ura4*$^{+}$ *GFP-bgs4:leu1*$^{+}$ *ade6-M210 h*$^{-}$	[7]
KGY2350-2	*bgs3Δ::ura4*$^{+}$ *GFP-bgs3:leu1*$^{+}$ *ura4-D18 his3-1 h*$^{+}$	[8]
**S2 Figure**		
KGY3494-2	*wsc1Δ::kanMX6 ura4-D18 ade6-M210 leu1-32 h*$^{-}$	Lab stock
KGY3500-2	*css1-3 wsc1Δ::kanMX6 ura4-D18 ade6-M210 leu1-32 h*$^{+}$	This study
KGY17567	*rho2Δ::ura4*$^{+}$ *ura4-D18 ade6-M210 leu1-32 h*$^{-}$	Lab stock
KGY3418-2	*css1-3 rho2Δ::ura4*$^{+}$ *ura4-D18 ade6-M210 leu1-32 h*$^{-}$	This study
KGY3559	*pmk1Δ::ura4*$^{+}$ *ura4-D18 ade6-M210 leu1-32 h*$^{+}$	Lab stock
KGY1250-2	*css1-3 pmk1Δ::ura4*$^{+}$ *ura4-D18 ade6-M210 leu1-32 h*$^{-}$	This study
KGY5385	*rgf1Δ::ura4*$^{+}$ *ura4-D18 ade6-M21X leu1-32 h*$^{+}$	Lab stock
KGY3524-2	*css1-3 rgf1Δ::ura4*$^{+}$ *ura4-D18 ade6-M210 leu1-32 h*$^{+}$	This study
KGY17574	*pck1Δ::ura4*$^{+}$ *ura4-D18 ade6-M21X leu1-32 h*$^{+}$	Lab stock
KGY4649-2	*css1-3 pck1Δ::ura4*$^{+}$ *ura4-D18 ade6-M210 leu1-32 h*$^{+}$	This study
KGY1829-2	*pck2Δ::ura4*$^{+}$ *ura4-D18 ade6-M21X leu1-32 h*$^{+}$	Lab stock
KGY4675-2	*css1-3 pck2Δ::ura4*$^{+}$ *ura4-D18 ade6-M210 leu1-32 h*$^{-}$	This study
KGY17508	*mkh1Δ::ura4*$^{+}$ *ura4-D18 ade6-M21X leu1-32 h*$^{+}$	Lab stock
KGY4676-2	*css1-3 mkh1Δ::ura4+ ura4-D18 ade6-M210 leu1-32 h*$^{+}$	This study
KGY113765	*pek1Δ::kanMX6 ura4-D18 ade6-M21X leu1-32 h+*	Bioneer V3
KGY4677-2	*css1-3 pek1Δ::kanMX6 ura4-D18 ade6-M210 leu1-32 h*$^{-}$	This study
KGY19827	*wsc1-mNG:kanMX6 ade6-M210 ura4-D18 leu1-32 h*$^{-}$	This study
KGY14969	*wsc1-mNG:kanMX6 css1-3 ade6-M210 ura4-D18 leu1-32 h*$^{-}$	This study
**S3 Figure**		
KGY1213	*cut6-1 ade6-M210 ura4-D18 leu1-32 h*$^{+}$	This study
KGY1128-2	*cut6-1 css1-3 ade6-M210 ura4-D18 leu1-32 h*$^{-}$	This study
KGY3426-2	*sur2Δ::kanMX6 ade6-M210 ura4-D18 leu1-32 h*$^{+}$	Bioneer V3
KGY7675-2	*css1-3 sur2Δ::kanMX6 ade6-M210 ura4-D18 leu1-32 h*$^{+}$	This study
KGY5517-2	*lac1Δ::kanMX6 ade6-M210 ura4-D18 leu1-32 h*$^{-}$	This study
KGY5904-2	*lac1Δ::kanMX6 css1-3 ade6-M210 ura4-D18 leu1-32 h*$^{+}$	This study
KGY5467-2	*lag1Δ::kanMX6 ade6-M210 ura4-D18 leu1-32 h*$^{+}$	This study
KGY5509-2	*lac1Δ::kanMX6 css1-3 ade6-M210 ura4-D18 leu1-32 h-*	This study
**S4 Figure**		
KGY19736	*ura4*$^{+}$*:Pact1-mCherry-D4H h*$^{+}$	[1]
KGY19753	*css1-3 ura4*$^{+}$*:Pact1-mCherry-D4H ade6-M210 ura4-D18 leu1-32 h*$^{+}$	This study
KGY423-2	*erg4Δ::kanMX6 ade6-M210 leu1-32 h*$^{+}$	This study
KGY461-2	*erg4Δ::kanMX6 css1-3 ade6-M210 leu1-32 h*$^{+}$	This study
KGY3492-2	*erg5Δ::kanMX6 ura4-D18 ade6-M210 leu1-32 h*$^{-}$	This study
KGY3493-2	*erg5Δ::kanMX6 css1-3 ura4-D18 ade6-M210 leu1-32 h*$^{-}$	This study
**S5 Figure**		
KGY3692-2	*dnf2Δ::kanMX6 ura4-D18 ade6-M210 leu1-32 h*$^{-}$	This study
KGY3693-2	*dnf2Δ::kanMX6 css1-3 ura4-D18 ade6-M210 leu1-32 h*$^{-}$	This study
KGY3694-2	*dnf2.5Δ::kanMX6 ura4-D18 ade6-M210 leu1-32 h*$^{+}$	This study
KGY3695-2	*dnf2.5Δ::kanMX6 css1-3 ura4-D18 ade6-M210 leu1-32 h*$^{-}$	This study
KGY3866-2	*ppk14-mNG:kanMX6 ura4-D18 leu1-32 ade6-M210 h*$^{-}$	This study

KGY4666-2	*dnf1-moxNG:kanMX6 ura4-D18 leu1-32 ade6-M210 h*$^{-}$	This study
KGY3667-2	*slm1-mNG:kanMX6 efr3Δ::kanMX6 ura4-D18 ade6-M210 leu1-32 h*$^{-}$	This study
**S6 Figure**		
KGY5469-2	*gpi8-1:kanMX6 ade6-M210 ura4-D18 leu1-32 h*$^{-}$	This study
KGY5405-2	*ost1-3:kanMX6 ade6-M210 ura4-D18 leu1-32 h*$^{+}$	This study
KGY6295-2	*mok1-664 ghs2-2:kanMX6 ade6-M210 ura4-D18 leu1-32 h*$^{+}$	This study
KGY6363-2	*cps1-191 ghs2-2:kanMX6 ade6-M210 ura4-D18 leu1-32 h*$^{+}$	This study
KGY6296-2	*cwg1-1 ghs2-2:kanMX6 ade6-M210 ura4-D18 leu1-32 h*$^{+}$	This study

References

1. Marek M, Vincenzetti V, Martin SG. Sterol biosensor reveals LAM-family Ltc1-dependent sterol flow to endosomes upon Arp2/3 inhibition. J Cell Biol. 2020;219(6). Epub 2020/04/23. doi: 10.1083/jcb.202001147. PubMed PMID: 32320462; PubMed Central PMCID: PMCPMC7265315.
2. Bimbo A, Jia Y, Poh SL, Karuturi RK, den Elzen N, Peng X, et al. Systematic deletion analysis of fission yeast protein kinases. Eukaryotic cell. 2005;4(4):799-813. Epub 2005/04/12. doi: 10.1128/EC.4.4.799-813.2005. PubMed PMID: 15821139; PubMed Central PMCID: PMC1087820.
3. Curto MA, Sharifmoghadam MR, Calpena E, De Leon N, Hoya M, Doncel C, et al. Membrane organization and cell fusion during mating in fission yeast requires multipass membrane protein Prm1. Genetics. 2014;196(4):1059-76. Epub 2014/02/12. doi: 10.1534/genetics.113.159558. PubMed PMID: 24514900; PubMed Central PMCID: PMCPMC3982680.
4. Katayama S, Hirata D, Arellano M, P Pr, Toda T. Fission Yeast a-Glucan Synthase Mok1 Requires the Actin Cytoskeleton to Localize the Sites of Growth and Plays an Essential Role in Cell Morphogenesis Downstream of Protein Kinase C Function. J Cell Biol. 1999;144(6):1173-86.
5. Ribas JC, Diaz M, Duran A, Perez P. Isolation and characterization of Schizosaccharomyces pombe mutants defective in cell wall (1-3)beta-D-glucan. J Bacteriol. 1991;173(11):3456-62. Epub 1991/06/01. doi: 10.1128/jb.173.11.3456-3462.1991. PubMed PMID: 1828464; PubMed Central PMCID: PMCPMC207959.
6. Cortes JC, Sato M, Munoz J, Moreno MB, Clemente-Ramos JA, Ramos M, et al. Fission yeast Ags1 confers the essential septum strength needed for safe gradual cell abscission. J Cell Biol. 2012;198(4):637-56. doi: 10.1083/jcb.201202015. PubMed PMID: 22891259; PubMed Central PMCID: PMCPMC3514033.
7. Cortes JC, Carnero E, Ishiguro J, Sanchez Y, Duran A, Ribas JC. The novel fission yeast (1,3)beta-D-glucan synthase catalytic subunit Bgs4p is essential during both cytokinesis and polarized growth. J Cell Sci. 2005;118(Pt 1):157-74. PubMed PMID: 15615781.
8. Cortes JC, Konomi M, Martins IM, Munoz J, Moreno MB, Osumi M, et al. The (1,3)beta-D-glucan synthase subunit Bgs1p is responsible for the fission yeast primary septum formation. Mol Microbiol. 2007;65(1):201-17. Epub 2007/06/22. doi: 10.1111/j.1365-2958.2007.05784.x. PubMed PMID: 17581129.
